# Supplementary material for: How do cohabitation and marital status affect mortality risk? Results from a cohort study in Thailand
Source: BMJ Open. 2022 Sep 19;12(9):e062811. doi: 10.1136/bmjopen-2022-062811 (PMC9486337; doi:10.1136/bmjopen-2022-062811)
Supplement: Supplementary data [file bmjopen-2022-062811supp001.pdf]

Appendix

Appendix Table 1 Union status (2005) §, family support (2005), and Mortality (2005–2016) by family support in the Thai Cohort Study, 2005-2016

Model§		Union Status							
		Single	cohabited and living with partner	Cohabit but not living with partner	Married but not living together	Married and living together	Separated, divorce or widowed	Separated, Divorced, widowed, but living with partner	Not Having Good Family Support
Model 2		1.36(1.17-1.57)***	1.46(1.12-1.90)**	0.95(0.52-1.74)	1.35(1.03-1.76)*	Reference	1.65(1.31-2.08)***	1.39(0.87-2.22)	1.39(1.19-1.62)***
Model 2.1		1.31(1.12-1.53)***	1.64(1.24-2.17)***	0.81(0.40-1.64)	1.32(0.99-1.77)	Reference	1.49 (1.13-1.98)**	1.57 (0.92-2.67)	0.85(0.28-2.58)
	Interaction term	1.83 (0.59-5.70）	0.49（0.11-2.21）	3.33 (0.59-18.81)	1.72(0.46-6.24)	1.49 (0.47-4.67）	2.15(0.65-7.11)	--	--
Model 2.2		1.31(1.12-1.53)***	1.64(1.24-2.17)***	0.81(0.40-1.63)	1.32(0.98-1.76)	Reference	1.50 (1.14-1.99)***	1.58 (0.93-2.68)	--
Model 2.3		1.61(1.12-2.34)*	0.55(0.20-1.55)	1.91(0.59-6.19)	1.60(0.79-3.24)	Reference	2.09(1.34-3.26)***	1.09 (0.40-3.01)	--

§Model 2: included union status+family support+age+sex+urban

Model 2.1: included union status+family support+age+sex+urban+union status*family support

Model 2.2 for those with good family support only: included union statust+age+sex+urban

Model 2.3 for those without good family support only: included union statust+age+sex+urban

* p<0.05

**p<0.01

***p<0.005

Appendix Table 2 Union status (2005) §, family support (2005), and Mortality (2005–2016) by age group in the Thai Cohort Study, 2005-2016

Birth year		Number of cases included in the model (% of total sample by sex)	Union Status						Not Having Good Family Support
			Single	cohabited and living with partner	Cohabit but not living with partner	Married but not living together	Separated, divorce or widowed	Separated, Divorced, widowed, but living with partner	
-1974		84874 (97.4%)	1.40(1.21-1.61)***	1.50(1.15-1.96)***	0.98(0.53-1.78)	1.37(1.05-1.79)*	1.73(1.38-2.18)***	1.46(0.92-2.34)	--
1975-		35181(90.9%)	1.17(0.98-1.43)	1.18(0.77-1.81)	1.60(0.71-3.59)	1.33(0.97-1.82)	1.45(1.12-1.89)***	1.10(0.63-1.91)	1.38(1.14-1.68)***
Total		44104(91.1%)	1.31(0.98-1.74)	1.54(1.02-2.31)*	0.64(0.26-1.60)	0.78(0.34-1.81)	1.01(0.40-2.52)	2.58(0.62-10.60)	1.31(0.99-1.75)

Models included union status+family support+age+sex+urban+income+ health behaviour +doctor diagnosed depression or anxiety+pre-existing physical conditions

§Reference group: married people and living together

* p<0.05
**p<0.01
***p<0.005

Appendix Table 3 Number of death by cause of death and union status in the Thai Cohort Study, 2005- 2010

Cause of Death*	Union Status								
	Single	cohabited and living with partner	Cohabit but not living with partner	Married but not living together	Married living together	Separated, divorce or widowed	Separated, Divorced, widowed, but living with partner	Missing	Total
Cardiovascular disease	29 (37.18%)	5 (6.41%)	0 (0%)	7 (8.97%)	33 (42.31%)	2 (2.56%)	0 (0%)	2 (2.56%)	78 (100%)
Neoplasms	33 (27.97%)	1（0.85%)	0 (0%)	8 (6.78%)	57 (48.31%)	15 (12.71%)	1 (0.85%)	3 (2.54%)	118 (100%)
Injury	107 (52.45%)	12 (5.88%)	3 (1.47%)	4 (1.96%)	67 (32.84%)	10 (4.90%)	0 (0%)	1 (0.49%)	204(100%)
Other	78 (42.62%)	5 (2.73%)	2 (1.09%)	11 (6.01%)	69 (37.70%)	8 (4.37%)	3 (1.64%)	7 (3/83%)	183 (100%)

*Cardiovascular disease (CVD) (ICD code = I00-I99), Neoplasms (ICD code = C00-D48), injury (ICD code = V01-Y98), and from “other causes of death”
