# Supplementary material for: How do cohabitation and marital status affect mortality risk? Results from a cohort study in Thailand
Source: BMJ Open. 2022 Sep 19;12(9):e062811. doi: 10.1136/bmjopen-2022-062811 (PMC9486337; doi:10.1136/bmjopen-2022-062811)
Supplement: Author's manuscript [file bmjopen-2022-062811.draft_revisions.pdf]

BMJ Open

BMJ Open is committed to open peer review. As part of this commitment we make the peer review history of every article we publish publicly available.

When an article is published we post the peer reviewers' comments and the authors' responses online. We also post the versions of the paper that were used during peer review. These are the versions that the peer review comments apply to.

The versions of the paper that follow are the versions that were submitted during the peer review process. They are not the versions of record or the final published versions. They should not be cited or distributed as the published version of this manuscript.

BMJ Open is an open access journal and the full, final, typeset and author-corrected version of record of the manuscript is available on our site with no access controls, subscription charges or pay-per-view fees (http://bmjopen.bmj.com).

If you have any questions on BMJ Open's open peer review process please email info.bmjopen@bmj.com

BMJ Open

Cohabitation, marital status, mortality and gender difference in Thailand

Journal:	*BMJ Open*
Manuscript ID	bmjopen-2022-062811
Article Type:	Original research
Date Submitted by the Author:	28-Mar-2022

Complete List of Authors:	Zhao, Jiaying; Australian National University<br>Law, CK; The University of Sydney<br>Kelly, Matthew; Australian National University, Department of Global Health<br>Yiengprugsawan, Vasoontara; University of New South Wales<br>Seubsman, Sam-ang; Sukhothai Thammathirat Open University<br>Sleigh, Adrian; Australian National University, National Centre for Epidemiology and Population Health

Keywords:	PUBLIC HEALTH, EPIDEMIOLOGY, SOCIAL MEDICINE

SCHOLARONE™
Manuscripts

I, the Submitting Author has the right to grant and does grant on behalf of all authors of the Work (as defined in the below author licence), an exclusive licence and/or a non-exclusive licence for contributions from authors who are: i) UK Crown employees; ii) where BMJ has agreed a CC-BY licence shall apply, and/or iii) in accordance with the terms applicable for US Federal Government officers or employees acting as part of their official duties; on a worldwide, perpetual, irrevocable, royalty-free basis to BMJ Publishing Group Ltd ("BMJ") its licensees and where the relevant Journal is co-owned by BMJ to the co-owners of the Journal, to publish the Work in this journal and any other BMJ products and to exploit all rights, as set out in our licence.

The Submitting Author accepts and understands that any supply made under these terms is made by BMJ to the Submitting Author unless you are acting as an employee on behalf of your employer or a postgraduate student of an affiliated institution which is paying any applicable article publishing charge ("APC") for Open Access articles. Where the Submitting Author wishes to make the Work available on an Open Access basis (and intends to pay the relevant APC), the terms of reuse of such Open Access shall be governed by a Creative Commons licence – details of these licences and which Creative Commons licence will apply to this Work are set out in our licence referred to above.

Other than as permitted in any relevant BMJ Author's Self Archiving Policies, I confirm this Work has not been accepted for publication elsewhere, is not being considered for publication elsewhere and does not duplicate material already published. I confirm all authors consent to publication of this Work and authorise the granting of this licence.

For peer review only

Cohabitation, marital status, mortality and gender difference in Thailand

Jiaying Zhao[a], Chi Kin Law[b], Matthew Kelly[c§],Vasoontara Yiengprugsawan[d] , Sam-ang Seubsman[f], Adrian Sleigh[c]

[a]School of Demography, The Australian National University, Canberra, ACT, Australia 2601

[b]NHMRC Clinical Trials Centre, Faculty of Medicine and Health, The University of Sydney, Australia

[c]Department of Global Health, Research School of Population Health, The Australian National University, Australia

[d]Australian Research Council Centre of Excellence on Population Ageing Research, Business School, University of New South Wales, Australia

[f]School of Human Ecology, Sukhothai Thammathirat Open University, Pakkret, Nonthaburi, Thailand

[§] **Correspondence to:** Dr Matthew Kelly, 62 Mills Rd., The Australian National University, Acton 2601, Canberra, Australia. +61 2 6125 0714

Email Matthew.Kelly@anu.edu.au

Word count: 3921

For peer review only

Funding statement

This study was supported by the International Collaborative Research Grants Scheme with joint grants from the Wellcome Trust UK (GR071587MA) and the Australian NHMRC (268055) and by a global health grant from the NHMRC (585426).

Ethical approval

Informed written consent was obtained from all participants. All students were advised that they could withdraw, or not participate, without any effect on their academic progress. The questionnaires never sought sensitive personal information and no biological samples were taken. Ethics approval was obtained from Sukhothai Thammathirat Open University Research and Development Institute (protocol 0522/10) and the Australian National University Human Research Ethics Committee (protocols 2004/344 and 2009/570).

Data availability statement

Data from the Thai Cohort Study are available upon reasonable request from the corresponding author (MK).

Acknowledgements

We thank the staff at Sukhothai Thammathirat Open University (STOU) who assisted with student contact and the STOU students who are participating in the cohort study. We also thank Dr Bandit Thinkamrop and his team from Khon Kaen University for guiding us successfully through the complex data processing.

For peer review only

For peer review only - http://bmjopen.bmj.com/site/about/guidelines.xhtml

Competing interests

All authors declare no conflict of interest in this paper

For peer review only

For peer review only - http://bmjopen.bmj.com/site/about/guidelines.xhtml

Cohabitation, marital status, mortality and gender difference in Thailand

Abstract

Objective

To examine the relationship between baseline union status (i.e. including marriage and cohabitation) and mortality, paying attention to gender differentials, through an 11-year follow-up of a large cohort in Thailand.

Design

Cohort data from Thai Cohort Study (TCS) were linked official death records over an 11 year follow-up period.

Setting

Community based adults in Thailand

Participants

87,151 Thai adults participated in TCS cohort.

Method Cox regression models measured longitudinal associations between union status and 11-year-mortality.

Results

For peer review only - http://bmjopen.bmj.com/site/about/guidelines.xhtml

From 2005 (baseline) to 2016, persons who cohabited and lived with a partner, married persons but not living with a partner, and separated /divorced/widowed people were more likely to die compared with those married and living together with a partner. Those who did not have good family support had a higher death risk than those having good family support.

Single or cohabiting women had higher risks of mortality than women who were married and living together with a partner throughout follow-up, while separated/divorced/widowed men had higher risks of mortality than counterpart males.

Conclusions

Our study reveals the protective effect of marriage and living together on mortality in Thailand, an understudied setting where institutionalization of cohabitation is low leading to a limited mortality protection. Public policies for moderating mortality should thus be gender nuanced, culturally and institutionally specific. Also, we demonstrate that in settings such as Thailand, where marital status is not always defined in the same way as in western cultures, the need to measure cohabitation in locally relevant terms is important.

Keywords: gender difference, cohabitation, marriage, mortality, Thailand

For peer review only - http://bmjopen.bmj.com/site/about/guidelines.xhtml

For peer review only

For peer review only - http://bmjopen.bmj.com/site/about/guidelines.xhtml

Strengths and Limitations of the study

- This is a large nationwide cohort (i.e. 87,151 participants) with 11-year follow-up period produced reliable estimate of effect sizes while also measuring the longitudinal effects of changes in union status on mortality.

- The study filled the gap by examining whether cohabitation affords Eastern populations' protective benefits similar to marriage, taking living arrangement into account. The potential mechanism linking union status to mortality from socioeconomic, psychological, and behavioural aspects was investigated.

- Although the Thai Cohort Study participants are not randomly representative in terms of educational attainment, selection bias when establishing the cohort should not interfere with relative risk estimates between exposed and unexposed individuals for the socio-biological outcome of death. HRs estimated from groups within a cohort remain valid and can be generalised more broadly.

- The difficulty in defining marriage and cohabitation in Thailand may indicate there was some misclassification of individuals making the cohabitation – mortality relationship difficult to measure.

For peer review only - http://bmjopen.bmj.com/site/about/guidelines.xhtml

Introduction

Inequalities of mortality risk across categories of marital status have been well documented by previous longitudinal studies [1-3]. Married persons generally have lower mortality than those who are never married, divorced, separated, or widowed in both developed [4] and developing countries [5]. Such patterns were found to be more marked among men than women [6, 7].

Two major processes have been proposed to explain the marital status differences in mortality risks: (1) selection mechanisms and; (2) protection mechanisms [1, 8]. Selection theory suggests that unhealthy people may be selected outside the bounds of marriage into other union types, such as cohabitation [9]. The 'Protection mechanisms' theory suggests that marital status has a causal impact on mortality risk reduction through the provision of social/ economic support, through improvement of unhealthy behaviours and mental health, and provision of health information [1, 3, 6, 10, 11]. A meta-analysis indicates that support from family was more beneficial than support provided by friends in reducing mortality [6]. A further clarification of the interaction between family support and union status and their effects on mortality can improve our understanding of the pathways between union status and mortality risk reduction.

Cohabitation may facilitate similar functions as marriage, though such a protection effect may be weaker than marriage and may vary across populations [9, 12, 13]. Some evidence from developed countries shows that cohabiting persons have lower mortality than those living alone, particularly for men and a combination of married and cohabited state may better predict mortality than marriage alone [13, 14]. Similar to married status, cohabitation may improve health through emotional and instrumental support from partners [15]. But cohabitation, with lower levels of commitment, support and stability than marriage, could constrain economic, social, and psychological resources [9, 15, 16]. Also, living arrangements, incorporating the partnership situation and household composition, was

For peer review only

For peer review only - http://bmjopen.bmj.com/site/about/guidelines.xhtml

associated with mortality [17, 18] . Evidence on the health effect of cohabitation, particularly incorporating living arrangements is inconsistent and sparse in developing countries.

Gender differences could be another potential moderator for the effect of union status on mortality [3, 14]. Men's mortality may profit more from union status than women [3, 13, 19, 20]. Men may benefit more from risk behaviour reduction and emotional support than women [19, 21], while women may benefit more financially [1, 22]. After union disillusion, excess mortality is greater among men than women [20, 23]. This may be because women are more likely to adopt the role of providing emotional support and performing household tasks for their spouse. Men may suffer more once union status dissolves [13] but separated/divorced/ widowed women may feel relieved of their emotional duty to care, and consequently lower their mortality [24].

Gender differences in the effect of union status on mortality may vary in societies with different gender role norms (e.g. the male breadwinner model) [24]. In societies with a low degree of institutionalization of cohabitation, the mortality benefit from union status due to financial support may be weaker for cohabited women than married women. Cohabited women may also benefit less emotionally from union status due to low social acceptance of cohabitation.

Cohabitation has become more common in Asian countries [25, 26]. In Thailand, the proportion of cohabiting individuals increased from 6% in 2005-2009 to 9% in 2010-2014 [27]. However, cohabitation may have different social, cultural and institutional interpretations as well as legal rights for relationships in Eastern societies.

Thai society has strong religious identification [28], and more traditional cultural norms around cohabitation [29]. The strong religious identification has a role in the production of gender norms and expectations [30]. However, during the past decades, attitudes towards marriage have been changing [31, 32] . Increased female workforce participation, work related pressures, high costs of living and

For peer review only - http://bmjopen.bmj.com/site/about/guidelines.xhtml

an increasing burden of care giving for elderly parents have led to delayed marriages and downward pressure on marriage rates [31, 33].

Reported cohabitation rates remained low in Thailand, however this may relate to the inexact definition of marriage in Thai society. State registration of marriage is practiced in Thailand but those married through traditional Buddhist religious ceremonies are also considered married. In 2005, around 34% of currently married people were not registered, and the proportion was higher for those aged 15-34 years [34]. Overall, cohabiting without marriage in Thailand is likely to be more common than surveys reveal [31]. Younger, more educated and Bangkok dwelling Thais are more likely to cohabit with their partner [25].

In many Western countries de facto partnering confers rights and obligations on partners that are identical to those married. Those rights include economic security and sharing of marital property in the event of divorce [35]. However, cohabitation in Thailand does not incur the same legal outcomes as registered marriages in terms of sharing property and other assets [36].

Variation in different social, cultural and institutional interpretations as well as legal rights for relationships may lead to different associations between cohabitation and mortality [6, 37, 38]. The gap in mental health between married and cohabitating individuals is larger in countries with a low degree of institutionalization of cohabitation. In such countries the following features are expected: 1) cohabitation is relatively uncommon and low in social acceptance [38]; 2) traditional gender role persists [37]; 3) and religiosity is high [37]. Disapproval of cohabitations and uncertainty of future may lead to lower level of social support and poorer mental wellbeing for cohabiters in such society [38].

Previous studies examined the influence of marital status on mortality in Eastern societies but did not differentiate between cohabitation and marriage [39]. It remains unclear whether cohabitation affords Eastern populations' protective benefits similar to marriage.

For peer review only

For peer review only - http://bmjopen.bmj.com/site/about/guidelines.xhtml

This study aims to fill this knowledge gap by examining the relationship between baseline union status (i.e. including marriage and cohabitation) and mortality, paying attention to gender differentials, through an 11-year follow-up of a large cohort in Thailand. The potential mechanism linking union status to mortality from socioeconomic, psychological, and behavioural aspects will be investigated.

Hypotheses

Considering the institutional and cultural features around marriage in Thailand, we test the following hypotheses:

1. People who were single, or separated/divorced/widowed have higher mortality than married people living together.

2. Cohabiting people living with a partner have a higher mortality risk than married people living together, but may have lower mortality than single, or separated/divorced/widowed people.

3. People who have stronger family support have lower mortality, and this effect cannot be fully explained by differentials in union status.

4. 'Separated/divorced/widowed' males have a higher mortality risk compared with married men living together with a partner, while such higher mortality risk may be moderated among females.

5. Cohabiting women living with a partner have a higher mortality risk compared with married women living together with a partner, while such higher mortality risk may be limited among males

Methods

Study population and study design

For peer review only

Data from a large nationwide longitudinal study of health dynamics in Thailand – the Thai Cohort Study (TCS) – were linked with official death records over an 11 year follow-up period. The baseline cohort was recruited in 2005 with a questionnaire mailed to all current students of Sukhothai Thammathirat Open University (STOU); 87,151 responded, covering 43.5% of the STOU students at that time. STOU students are off-campus adult distance-learners residing throughout the country. Characteristics of TCS members are similar to the overall Thai population, in terms of social geography, religion, socioeconomic status, and income, but members are in general more highly educated and more urban [40]. In 2005, at the study baseline, the cohort and the general Thai population shared a median age of 29 years, median income of around $2500USD, were 94% Buddhist, and just over 50% female, and had a similar regional distribution across the country [40, 41].

Measurements

Mortality

Mortality status was traced with death registration records from the Thai Ministry of Interior covering the period from TCS baseline in 2005 to November 2016. The current death registration system covers about 95% of all deaths in Thailand [42]. Data linkage was conducted by matching the unique citizen identification number, which cohort members provided to the study at baseline. By November 2016, 1401 deaths were recorded within the cohort.

Union status

Using the TCS baseline data, union status was categorised by the following three questions: 1. 'What is your current status?' (single, living with partner, or married). Those who responded 'Married' were further classified by question：2.'What is your current marital status?' Those who responded 'Married – first and only marriage' or 'Remarried – second or later marriage'

For peer review only - http://bmjopen.bmj.com/site/about/guidelines.xhtml

were classified as ‘Married’, while those who responded ‘Separated from someone you have been married to (but not divorced)’, ‘Divorced’, or ‘Widowed’ were classified as ‘Separated/divorced/widowed’. We further classified union status with living arrangement by 3. ‘Do any of the following people (i.e. Spouse / partner) usually live in your home?’

Conjugal union status was coded into seven categories: (1) Single, (2) cohabited and living with a partner, (3) cohabited but not living with a partner, (4) married but not living together with a partner, (5) married and living together with a partner (the reference group), (6) separated/divorced/widowed, and (7) separated/divorced/widowed but living with a partner.

Family support

Family support was assessed by the following question using a four-point scale: ‘How would you rate the support you are getting from your family?’ Those who responded ‘very little’, ‘little’, or ‘Not relevant’ were coded as not having good family support, while those who responded ‘quite a bit of support’, and ‘a lot of support’ were coded as having good family support.

Other baseline covariates

A wide range of covariates were available including baseline variables describing sex, birth year, urban or rural residence, and income, smoking status and alcohol consumption at baseline, baseline pre-existing psychological and physical health conditions and baseline BMI. Details of each covariate are shown in Table 1.

We also tested social support in the model. Social support was measured using the question ‘How would you rate the support you are getting from the following: government, religious groups, and good friends/neighbours/ people in workplace’. Those who responded ‘very little’ or ‘little’, ‘not relevant’ were classified as not having good social support for that question. But

For peer review only - http://bmjopen.bmj.com/site/about/guidelines.xhtml
For peer review only

these covariates were not significantly associated with mortality; and we did not report it in the final result.

For peer review only

For peer review only - http://bmjopen.bmj.com/site/about/guidelines.xhtml

Table 1 The measurements and classification of *baseline (2005) covariates*

Variable	Measurement Question	Original response category	Analytical category
Sex	You are:-	Male; Female	Male; Female
Birth year	In what year were you born?	Year of birth	-1959; 1960-1969; 1970-1974; 1975-1979; 1980-
Residence	Where is your current permanent home located now?	Countryside; City/Town	Urban; Rural
Income	What is your average personal monthly income?	less than 3,000 Baht; 3,001-7,000 Baht;<br>7,001-10,000 Baht; 10,001-20,000 Baht ; 20,001-30,000 Baht;<br>over 30,000 Baht	Personal monthly income: ≤7,000, 7001-20,000, or >20,000 Baht (1 $US ~ 30 Baht)
Smoking status	1.Have you ever smoked?;<br>2. If you have **quit** smoking, at what age did you stop?	1. Yes; No (will categorised 'Never').<br>2. XX years; still smoking (will be categorised as 'current smoker'), otherwise will be categorised as 'former smoker',	Never; Former smoker; Current smoker
Alcohol consumption	Have you ever drunk alcohol?	Occasional social drinker; No, never;<br>Current regular drinker;<br>Used to drink before, now	Never drinker; Occasional social drinker; current regular drinker; former drinker

For peer review only - http://bmjopen.bmj.com/site/about/guidelines.xhtml

		stopped	
Pre-existing physical conditions	The next questions ask about some health conditions whether you ever been told by a doctor that you have this condition. (please tick all that apply)	Diabetes (needing insulin); Diabetes (do not need insulin); Liver disease (not cancer); Kidney disease; High cholesterol/high blood lipids; High blood pressure; Ischemic (coronary) heart disease; Cerebrovascular disease (stroke); liver cancer; lung cancer; Cancer of the digestive system; Breast cancer; Other cancers	Whether a doctor had ever diagnosed: diabetes, cancer, cardiovascular diseases (CVD), kidney and liver diseases
Pre-existing psychological conditions	The next questions ask about some health conditions whether you ever been told by a doctor that you have this condition. (please tick all that apply)	Depression / anxiety	Whether a doctor had ever diagnosed psychological conditions (i.e. depression or anxiety)
Body mass index (BMI)	1.What is your weight?<br>2. What is your height without shoes?	1. XXX kilograms<br>2. XXX cm	Calculated by validated self-reported height and weight (Lim et al. 2009). Asian BMI standards: underweight (<18.5), normal (18.5 to <23), overweight at risk (23 to <25), obese I (25 to <30), and obese II (≥30) (Kanazawa et al. 2002)

For peer review only

Statistical analyses

Our longitudinal analyses of all-cause mortality continued until the end of the observation period in November 2016. The exposure variables of interest were union status and family support. We tabulated November 2016 survival status by union status, family support and all covariates.

We examined links between mortality as the outcome and union status and family support as the mutually adjusted exposure variables of interest. We used Cox regression to estimate multivariate hazard ratios (HR) for men and women and their 95% confidence intervals (CI) given the assumption of proportionality of hazards were held. Covariates were included in sequence as models were developed incrementally.

To examine the effect of other covariates on the association between baseline union status and the subsequent mortality outcome, 4 regression models were compiled. The included covariates and the exposure variables of interest for each model are shown in Table 2. The variance inflation factors (VIF) for all covariates and the exposure variables were less than 4, indicating no multicollinearity issues in the models [43]. We further applied the above four models by sex.

Table 2 Variables included in the analysed models

Model	Variables included
1	baseline union status and demographic variables (i.e. sex, year of birth, residence)
2	variables in Model 1, and family support
3	variables in Model 2, and socio-economic variables (i.e. income)
4	variables in Model 3, and related health behaviours (i.e. smoking status, alcohol consumption), pre-existing psychological and physical conditions, and BMI

Patient and public involvement statement

Study participants were all enrolled students at an Open University in Thailand. Research questions and approaches were first piloted with these students and the study design was adjusted accordingly. At various stages through the research project participants were provided with

For peer review only - http://bmjopen.bmj.com/site/about/guidelines.xhtml

summary results of the study. As well the lead investigator made frequent public appearances on University radio and TV media to discuss the results of the study.

Results

At 2005 baseline, 49.3% of cohort members were single, 4.9% were cohabited and living with a partner, 1.4% were cohabited but not living with a partner, 3% were married but not living with a partner, 35.3% were married living together with a partner, and 3.7% were separated /divorced/widowed and not living with a partner, 0.6% were separated /divorced/widowed but living with a partner . There were 89.7% members reporting have good family support. The relationship between baseline union status, family support, and a range of potential confounders were shown in Table 3.

For peer review only

Table 3 *Socio-demographic attributes at baseline (2005) by union status in the Thai Cohort Study*

		Single (%)		Cohabited and living with a partner (%)		Cohabited but not living with a partner (%)		Married but not living with a partner (%)		Married and living with a partner (%)		Separated/ divorced/ widowed (%)		Separated/ divorced/ widowed but living with a partner (%)		Missing (%)		Total (%)	
Total		42931		4235		1201		2624		30721		3206		566		1667		87151	
Sex	Males	16649	38.8	1983	46.8	517	43.0	1340	51.1	16759	54.6	1086	33.9	345	61.0	806	48.4	39485	45.3
	Females	26282	61.2	2252	53.2	684	57.0	1284	48.9	13962	45.4	2120	66.1	221	39.0	853	51.2	47658	54.7
	Missing	0	0.0	0	0.0	0	0.0	0	0.0	0	0.0	0	0.0	0	0.0	8	0.5	8	0.0
Birth year	-1959	584	1.4	48	1.1	10	0.8	304	11.6	3799	12.4	460	14.3	107	18.9	74	4.4	5386	6.2
	1960-1969	3404	7.9	399	9.4	76	6.3	732	27.9	11596	37.7	1353	42.2	260	45.9	217	13.0	18037	20.7
	1970-1974	5248	12.2	715	16.9	149	12.4	610	23.2	7454	24.3	735	22.9	120	21.2	243	14.6	15274	17.5
	1975-1979	12604	29.4	1434	33.9	464	38.6	680	25.9	5831	19.0	513	16.0	60	10.6	423	25.4	22009	25.3
	1980-	21088	49.1	1639	38.7	502	41.8	298	11.4	2035	6.6	144	4.5	19	3.4	702	42.1	26427	30.3
	Missing	3	0.0	0	0.0	0	0.0	0	0.0	6	0.0	1	0.0	0	0.0	8	0.5	18	0.0
Residence	Urban	21571	50.2	2486	58.7	700	58.3	1241	47.3	15867	51.6	1732	54.0	359	63.4	808	48.5	44764	51.4
	Rural	21098	49.1	1729	40.8	495	41.2	1359	51.8	14621	47.6	1427	44.5	199	35.2	820	49.2	41748	47.9
	Missing	262	0.6	20	0.5	6	0.5	24	0.9	233	0.8	47	1.5	8	1.4	39	2.3	639	0.7
Personal Income	-7000	23166	54.0	2053	48.5	541	45.0	818	31.2	6966	22.7	1019	31.8	140	24.7	933	56.0	35636	40.9
	7001-20000	16543	38.5	1810	42.7	587	48.9	1446	55.1	17513	57.0	1633	50.9	294	51.9	542	32.5	40368	46.3
	20001-	1927	4.5	275	6.5	50	4.2	321	12.2	5752	18.7	450	14.0	110	19.4	71	4.3	8956	10.3
	Missing	1295	3.0	97	2.3	23	1.9	39	1.5	490	1.6	104	3.2	22	3.9	121	7.3	2191	2.5
Smoking status	Never	33659	78.4	2863	67.6	809	67.4	1648	62.8	19035	62.0	2135	66.6	291	51.4	712	42.7	61152	70.2
	Former	4950	11.5	732	17.3	224	18.7	570	21.7	7181	23.4	583	18.2	169	29.9	293	17.6	14702	16.9
	current	3550	8.3	567	13.4	148	12.3	293	11.2	3533	11.5	359	11.2	90	15.9	192	11.5	8732	10.0
	Missing	772	1.8	73	1.7	20	1.7	113	4.3	972	3.2	129	4.0	16	2.8	470	28.2	2565	2.9
Alcohol consumption	Occasional social drinker	24676	57.5	2648	62.5	831	69.2	1607	61.2	18463	60.1	1877	58.5	345	61.0	924	55.4	51371	58.9
	Never drinker	13181	30.7	902	21.3	182	15.2	559	21.3	6818	22.2	715	22.3	84	14.8	271	16.3	22712	26.1
	Current regular drinker	1243	2.9	251	5.9	65	5.4	144	5.5	2187	7.1	173	5.4	54	9.5	54	3.2	4171	4.8
	Now stopped	3416	8.0	404	9.5	114	9.5	267	10.2	2848	9.3	387	12.1	78	13.8	210	12.6	7724	8.9
	Missing	415	1.0	30	0.7	9	0.7	47	1.8	405	1.3	54	1.7	5	0.9	208	12.5	1173	1.3
Pre-existing psychological conditions	Not Having	41365	96.4	4083	96.4	1157	96.3	2546	97.0	29875	97.2	3013	94.0	536	94.7	1585	95.1	84160	96.6
	Having	1566	3.6	152	3.6	44	3.7	78	3.0	846	2.8	193	6.0	30	5.3	82	4.9	2991	3.4

For peer review only - http://bmjopen.bmj.com/site/about/guidelines.xhtml

Union status, family support, and mortality

From 2005 (baseline) to 2016, separated /divorced/widowed people and married not living with a partner were more likely to die than married people living with a partner , but singles and those who cohabited were less likely to die compared to married people living together with a partner (Table 4). Those not having good family support were more likely to die than those having family support. Males, older birth cohorts, those living in rural areas, current smokers, current regular drinkers, those having psychological and chronic physical disease, and obese people were more likely to die.

For peer review only

For peer review only - http://bmjopen.bmj.com/site/about/guidelines.xhtml

Table 4. Survival status (%) by sex, union status, family support, and potential confounders for the Thai Cohort Study, 2005-2016

		Males		Females		Total	
		Survival Status on December 201					
		Alive (%)	Dead (%)	Alive (%)	Dead (%)	Alive (%)	Dead (%)
	Number of death	38507	978	47235	423	85750	1401
Birth year	-1959	93.0	7.0	97.9	2.1	94.6	5.4
	1960-1969	97.3	2.7	98.6	1.4	97.9	2.1
	1970-1974	98.1	1.9	99.1	0.9	98.6	1.4
	1975-1979	98.3	1.7	99.3	0.7	98.9	1.1
	1980-	98.2	1.8	99.4	0.6	99.0	1.0
	Missing	100.0	0.0	100.0	0.0	100.0	0.0
Residence	Urban	97.7	2.3	99.1	0.9	98.5	1.5
	Rural	97.3	2.7	99.1	0.9	98.3	1.7
	Missing	95.7	4.3	99.4	0.6	97.7	2.3
Personal Income	-7000	97.6	2.4	99.2	0.8	98.6	1.4
	7001-20000	97.7	2.3	99.1	0.9	98.4	1.6
	20001-	97.2	2.8	98.8	1.2	97.9	2.1
	Missing	95.1	4.9	98.9	1.1	97.2	2.8
Smoking status	Never	98.2	1.8	99.1	0.9	98.9	1.1
	Former	97.3	2.7	99.0	1.0	97.6	2.4
	current	96.4	3.6	97.5	2.5	96.5	3.5
	Missing	96.4	3.6	99.1	0.9	98.1	1.9
Alcohol consumption	Occasional social drinker	97.9	2.1	99.2	0.8	98.5	1.5
	Never drinker	97.8	2.2	99.1	0.9	98.8	1.2
	Current regular drinker	96.6	3.4	98.1	1.9	96.7	3.3
	Now stopped	95.8	4.2	98.8	1.2	97.1	2.9
	Missing	96.2	3.8	99.4	0.6	98.2	1.8
Pre-existing psychological conditions	Not Having Psychological Diseases	97.6	2.4	99.1	0.9	98.4	1.6
	Having Psychological Diseases	96.0	4.0	98.7	1.3	97.6	2.4
Pre-existing physical conditions	Not having diabetes	97.6	2.4	99.1	0.9	98.5	1.5

For peer review only - http://bmjopen.bmj.com/site/about/guidelines.xhtml

	Having diabetes	90.5	9.5	94.6	5.4	91.7	8.3
	Not having cancer	97.5	2.5	99.1	0.9	98.4	1.6
	Having cancer	94.0	6.0	95.9	4.1	95.2	4.8
	Not Having CVD	97.6	2.4	99.1	0.9	98.4	1.6
	Having CVD	92.7	7.3	96.6	3.4	94.4	5.6
	Not Having Kidney/liver Diseases	97.6	2.4	99.2	0.8	98.5	1.5
	Having Kidney/liver Diseases	96.3	3.7	98.0	2.0	97.0	3.0
BMI	Underweight(<18.5)	97.3	2.7	99.2	0.8	98.9	1.1
	Normal (18.5 to <23)	97.7	2.3	99.2	0.8	98.6	1.4
	Overweight at risk (23 to <25)	97.8	2.2	98.9	1.1	98.2	1.8
	Obese I (25 to <30)	97.3	2.7	98.8	1.2	97.8	2.2
	Obese II (≥30)	96.5	3.5	97.9	2.1	97.2	2.8
	Missing	94.9	5.1	98.5	1.5	96.5	3.5
**Having good family support**	Yes	97.6	2.4	99.2	0.8	98.5	1.5
	No	96.5	3.5	98.6	1.4	97.5	2.5
	Missing	96.9	3.1	99.6	0.4	98.2	1.8
**Union status**	Single	98.0	2.0	99.2	0.8	98.7	1.3
	Cohabited and living with a partner	97.9	2.1	98.9	1.1	98.5	1.5
	Cohabited but not living with a partner	98.5	1.5	99.6	0.4	99.1	0.9
	Married but not living with a partner	96.7	3.3	98.7	1.3	97.7	2.3
	Married and living with a partner	97.3	2.7	99.1	0.9	98.1	1.9
	Separated/divorced/widowed	94.3	5.7	98.8	1.2	97.3	2.7
	Separated/divorced/widowed but living with a partner	95.7	4.3	98.2	1.8	96.6	3.4
	Missing	97.3	2.7	98.7	1.3	98.0	2.0

For peer review only

In the Cox proportional hazard model (Table 5), after controlling for sex, birth cohort, rural or urban residence, singles, persons who cohabited and living with a partner, married persons but not living with a partner, and separated /divorced/widowed people were more likely to die compared with those married living together with a partner (Model 1). Further adjustment of family support (Model 2) reduced the corresponding HR. Those who do not have good family support had a higher death risk than those having good family support. Overall, no significant interaction was found between union status and family support (p>0.05), except for those who cohabited and were living with a partner. Among respondents who had poor family support, no mortality risk difference was found in those cohabited and living with a partner (p=0.04).

For peer review only - http://bmjopen.bmj.com/site/about/guidelines.xhtml

Table 5. *Gender difference, Union status (2005)* [§]*, family support (2005), and Mortality (2005–2016) in the Thai Cohort Study, 2005-2016*

	Sex	Number of cases included in the model (% of total sample by sex)	Marital Status						Not Having Good Family Support
			Single	cohabited and living with partner	Cohabit but not living with partner	Married but not living together	Separated, divorce or widowed	Separated, Divorced, widowed, but living with partner	
1		84874 (97.4%)	1.40(1.21-1.61)***	1.50(1.15-1.96)***	0.98(0.53-1.78)	1.37(1.05-1.79)*	1.73(1.38-2.18)***	1.46(0.92-2.34)	--
2		84444 (96.9%)	1.36(1.17-1.57)***	1.46(1.12-1.90)**	0.95(0.52-1.74)	1.35(1.03-1.76)*	1.65(1.31-2.08)***	1.39(0.87-2.22)	1.39(1.19-1.62)***
3		82461 (94.6%)	1.28(1.11-1.49)***	1.43(1.09-1.87)*	0.95(0.52-1.73)	1.27(0.97-1.67)	1.48(1.16-1.88)***	1.16(0.70-1.95)	1.38(1.18-1.61)***
4	Total	79286 (91.0%)	1.27(1.09-1.48)***	1.39(1.06-1.83)*	0.96(0.52-1.76)	1.24(0.92-1.66)	1.46(1.13-1.87)***	1.22(0.73-2.05)	1.35(1.15-1.59)***
1		38390 (97.2%)	1.36(1.13-1.63)***	1.34(0.96-1.87)	1.05(0.52-2.12)	1.32(0.97-1.80)	2.18(1.67-2.85)***	1.39(0.82-2.37)	--
2		38169 (96.7%)	1.32(1.09-1.58)***	1.30(0.93-1.82)	1.03(0.51-2.09)	1.29(0.94-1.77)	2.09(1.59-2.74)***	1.33(0.78-2.27)	1.30(1.08-1.57)**
3		37325 (94.5%)	1.20(0.99-1.45)	1.23(0.88-1.73)	0.99(0.49-2.01)	1.19(0.86-1.64)	1.87(1.41-2.48)***	1.04(0.57-1.90)	1.30(1.08-1.57)**
4	Males	35907 (90.9%)	1.17(0.95-1.42)	1.17(0.83-1.65)	0.99(0.49-2.02)	1.14(0.80-1.62)	1.79(1.33-2.40)***	1.11(0.61-2.03)	1.29(1.06-1.57)**
1		46484 (97.5%)	1.42(1.11-1.81)**	1.87(1.19-2.94)**	0.81(0.26-2.58)	1.51(0.90-2.54)	1.12(0.72-1.73)	1.78(0.66-4.82)	--
2		46275 (97.1%)	1.39(1.09-1.78)**	1.80(1.15-2.82)*	0.79(0.25-2.49)	1.49(0.88-2.50)	1.07(0.69-1.66)	1.65(0.61-4.47)	1.60(1.21-2.10)***
3		45136 (94.7%)	1.40(1.10-1.80)**	1.87(1.19-2.93)**	0.83(0.26-2.63)	1.52(0.90-2.56)	0.98(0.62-1.55)	1.70(0.63-4.61)	1.56(1.18-2.06)***
4	Females	43379 (91.0%)	1.40(1.08-1.81)**	1.87(1.19-2.96)**	0.88(0.28-2.78)	1.49(0.87-2.56)	0.99(0.62-1.59)	1.75(0.64-4.75)	1.52(1.14-2.03)***

Note:

Model 1 included union status+age+sex+urban (family support not included)
Model 2 included union status+family support+age+sex+urban
Model 3 included union status+family support+age+sex+urban+income
Model 4 included union status+family support+age+sex+urban+income+ health behaviour +doctor diagnosed depression or anxiety+pre-existing physical conditions

For peer review only - http://bmjopen.bmj.com/site/about/guidelines.xhtml

For peer review only

§Reference group: married people
* $p<0.05$
**$p<0.01$
***$p<0.005$

For peer review only

For peer review only - http://bmjopen.bmj.com/site/about/guidelines.xhtml

When the effect of personal income was included (Model 3), the HR reduced but remained significant for singles, cohabited and living with a partner, and separated /divorced/widowed people, but the HR for those married but not living with a partner became not significant. HR for family support did not change significantly when income was included. When health behaviours, pre-existing psychological conditions and chronic conditions (Model 4) were included, the HR for union status and family support changed little.

In the final Model 4 certain covariates also associated with mortality risk were included. Males, older birth cohort members, rural residents, persons with low income, smokers, current regular drinkers or those who stopped drinking, and those having psychological or physical conditions were all more likely to die, compared with their counterparts (Appendix Table 1)

Sex differentials in the effect of union status on mortality

We repeated the models by sex with results shown in Table 5. Males and females had a different mortality differential pattern by union status. After controlling for birth cohort, rural or urban residence, both single men and women had higher risks of dying than their counterparts who have married living together with a partner. However, cohabited women living with a partner had higher risks of dying than married women living together with a partner, while such increase in mortality risk was not significant among men. By contrast, separated /divorced/widowed men had a higher mortality risk than counterpart men, while such risk did not significantly increase among separated /divorced/widowed women (Model 1). Further adjustment for family support (Model 2) reduced the corresponding HR. The HR for those not having good family support for females was higher than males.

When income was included in Model 3, the HRs for single and separated /divorced/widowed men reduced, but remained similar for single women. The HRs for cohabited women living with a partner was similar to those in Model 2, while those for men became not significant. HR for family

For peer review only

For peer review only - http://bmjopen.bmj.com/site/about/guidelines.xhtml

support did not change significantly when income was included. In the final Model 4, when health behaviours, pre-existing psychological and chronic conditions and BMI were included HR for union status and family support changed little in these models.

In the final Model 4, single women, cohabited women living with a partner, and separated/divorce/widowed men had significantly higher mortality risks than their married counterparts living together with a partner.

Discussion

The analyses add to Eastern population evidence on links between union status and mortality risk, as well as considering the under-explored factor of cohabitation. Single or cohabiting women had higher risks of mortality than women who were married and living together with a partner throughout follow-up. However, such increasing risk for single or cohabiting men was moderate. Separated/divorce/widowed men also had higher risks of mortality than men who were married and living together with a partner. Family support acts as a protective factor against mortality but such protective effect cannot fully be attributed to union status. These results confirm most of the hypotheses we proposed, except that cohabiting people do not appear to experience any protective effect over those who were single. In regard to the protective effect of marriage on mortality, this analysis agrees with much of the literature, and with similar effect sizes. For example in a meta-analysis, relative risks for mortality were calculated for widowed (1.11), divorced/separated (1.16) and never married (1.11) individuals, respectively [1]. Other studies however have found that cohabiting has a similar protective effect to marriage, unlike this Thai study. In fact in several studies there was no independent effect found for marriage on mortality, the protective function appeared to result from living together in any union status [13, 14]. More complex analyses which take into account a range of factors around social interaction appear to predict mortality more accurately than models that use union status alone [39].

For peer review only

For peer review only - http://bmjopen.bmj.com/site/about/guidelines.xhtml

A possible explanation for this divergence in results, may lie with the particular cultural setting of Thailand, and the lack of studies in similar settings. Defining and measuring marital status and cohabitation rates has historically been quite difficult in Thailand Jones 2007. Buddhist society has not traditionally had a definite view on when couples are married and this has led to legal issues surrounding inheritance, property and alimony. Generally a man's obligation to treat a woman as his wife was defined through the payment of a dowry by the woman's family, without legal registration [44].

A Family Registration Act in 1935 attempted to formalise marriage registration [44]. However, in modern Thailand multiple combinations of religious ceremony and state registration are still generally recognised [33]; and the Thai census itself may classify as married individuals who have only performed ceremonial bonding [31]. This is pertinent to our study with the possibility that some of those who self-reported being married may in fact according to western definitions be living in a cohabiting relationship. In Thailand cohabiting remains legally distinguishable from marriage and does not appear to confer mortality benefit but this needs more focused research to explain our results.

A potential explanation of high mortality risk for single and cohabited women living with a partner may be a consequence of social stigmatisation towards single or cohabitation status [45, 46]. Social stigma of single and cohabited persons and consequent stress of not being married may adversely affect persons' wellbeing and health status, in a society with a high prevalence of marriage [45]. In a society where most individuals are married, unmarried individuals have been seen as deviant from social role expectations [45]. For women, a 'marriage package' even comes laden with more traditional expectations of and for women [47, 48]. Behaving against the norm may cause that single and cohabitated people evaluate their lives as more incomplete than married persons [45].

Results of our analysis showed that the mortality risk differentials for single males reduced and were no longer significant once income was included in the model. In contrast, the same adjustment

For peer review only

had a limited impact for women. There is some evidence that men's low economic position would deter union formation, and that effect was more influential in countries where gender roles are more traditional [49]. Low-income men who are unable to fulfil the role of breadwinner are not only less attractive for marriage [49], but their low socioeconomic status also leads to a higher risk of mortality [50]. Further, in a traditional society (like Thailand), low-income men who failed to meet the social norm of 'male breadwinner' may suffer from higher psychological distress [51].

The gender difference in effect of marriage dissolution is also noteworthy. Separated/divorced/widowed men had much higher mortality risk than women. This result has also been found in previous studies, with the differential reducing in older age groups; that is middle-aged divorced or widowed men have the highest relative mortality risk [6, 52]. One proposed mechanism for this differential is a greater reduction in social ties and social support experienced by men when marriages end [3]. With women being more responsible for maintaining friendship and family ties they are more capable to maintain these relationships after the marriage ends [3].

The analysis included living arrangement combined with union status. It showed those married but living separately with a partner generally have a higher risk than those were married and living together, though the effect is not statistically significant in the final model. It is possible that those who died during 2005-2016 were ill or at greater risks of mortality at baseline. There were 9353 participants (10.7% of cohort) reporting having physician's diagnosis of depression / anxiety, cancer, cardiovascular disease, diabetes, liver disease, or kidney diseases at baseline. We performed a sensitivity analysis by excluding these participants from the survival analysis (see Appendix Table 2) and found HRs for union status and family support were generally similar to the final model (Model 4), which has adjusted for participants' baseline disease profile.

There were 79,295 (91% among 87,151) cohort members aged from 20-44 years. A sensitivity analysis was conducted to compare the final model (Model 4) among those aged 20-44 years with the model weighted by nationally representative Thai population. The national data were derived

For peer review only - http://bmjopen.bmj.com/site/about/guidelines.xhtml

from the Health and Welfare Survey 2005 conducted and reported by the National Statistical Office (Thailand) [53]. The HRs for union status and family support between the original model and model weighted by the Thai population were generally similar (Appendix Table 3).

Our large nationwide cohort and extensive follow-up period produced reliable estimate of effect sizes while also measuring the longitudinal effects of changes in union status. Although the TCS participants are not randomly representative in terms of educational attainment, the majority Buddhist religion, geographical distribution, variance in socio-economic wellbeing, as well as their community embedded residences give a reasonable picture of union status and mortality in Thailand [40]. The TCS is also future focused, including aspirational self-motivated Thais with increasing levels of education. Furthermore, unlike cross-sectional studies, longitudinal cohort analyses are designed to investigate causal relationships between exposures and outcomes within the same group of cohort population over time. Selection bias when establishing the cohort should not interfere with relative risk estimates between exposed and unexposed individuals for the socio-biological outcome of death [54]. HRs estimated from groups within a cohort remain valid and can be generalised more broadly [54, 55]. In addition, the difficulty in defining marriage and cohabitation in Thailand may indicate there was some misclassification of individuals making the cohabitation – mortality relationship difficult to measure.

Conclusions

Our 11-year cohort study revealed the protective effect of marriage on mortality in transitional Southeast Asia, an understudied Eastern setting. But such a protective effect of cohabitation, particularly for women, was limited in the Thai context. The gender difference found in this analysis suggests that union status effect on mortality may be mediated by socio-cultural factors in a society, which deserves for further investigation. The need to measure marriage and cohabitation in institutionally and culturally relevant terms is important. Formulation and implementation of public policies for moderating mortality should be gender nuanced and culturally and institutionally

For peer review only - http://bmjopen.bmj.com/site/about/guidelines.xhtml

specific. The correct policy will respond to the current situation and encourage people to be aware of the potential effect of union status on health and wellbeing.

Abbreviations

BMI: Body mass index; CI: Confidence Interval; CVD: cardiovascular diseases; HR: hazard ratios; TCS: Thai Cohort Study.

For peer review only

Funding

This research was jointly funded by the Australian National Health and Medical Research Council (grant nos. 268055 and 585426) and the Wellcome Trust (GR071587MA)

Author Contributions

JZ and CKL conceived the study design and led data analysis, MK and VY led data interpretation and manuscript writing. AS and SS conceived the overall study and made substantial contributions to data interpretation and critically reviewing the manuscript. All authors contributed to review and approval of the final draft.

Conflict of interest

All authors declare they have no conflict of interest in the publication of this article

Data sharing statement

All data used in this study are available from the corresponding author on reasonable request.

For peer review only

References:

1. Manzoli L, Villari P, G MP, Boccia A: **Marital status and mortality in the elderly: a systematic review and meta-analysis**. *Soc Sci Med* 2007, **64**(1):77-94.
2. Lund R, Holstein BE, Osler M: **Marital history from age 15 to 40 years and subsequent 10-year mortality: a longitudinal study of Danish males born in 1953**. *Int J Epidemiol* 2004, **33**(2):389-397.
3. Shor E, Roelfs DJ, Bugyi P, Schwartz JE: **Meta-analysis of marital dissolution and mortality: reevaluating the intersection of gender and age**. *Soc Sci Med* 2012, **75**(1):46-59.
4. Ebrahim S, Wannamethee G, McCallum A, Walker M, Shaper AG: **Marital status, change in marital status, and mortality in middle-aged British men**. *Am J Epidemiol* 1995, **142**(8):834-842.
5. Hurt LS, Ronsmans C, Saha S: **Effects of education and other socioeconomic factors on middle age mortality in rural Bangladesh**. *J Epidemiol Community Health* 2004, **58**(4):315-320.
6. Shor E, Roelfs DJ, Yogev T: **The strength of family ties: A meta-analysis and meta-regression of self-reported social support and mortality**. *Social Networks* 2013, **35**(4):626-638.
7. Tatangelo G, McCabe M, Campbell S, Szoeke C: **Gender, marital status and longevity**. *Maturitas* 2017, **100**:64-69.
8. Joung IM, van de Mheen HD, Stronks K, van Poppel FW, Mackenbach JP: **A longitudinal study of health selection in marital transitions**. *Soc Sci Med* 1998, **46**(3):425-435.
9. Liu H, Reczek C: **Cohabitation and US adult mortality: An examination by gender and race**. *Journal of Marriage and Family* 2012, **74**(4):794-811.
10. Cheung YB: **Marital status and mortality in British women: a longitudinal study**. *Int J Epidemiol* 2000, **29**(1):93-99.
11. Murphy M, Glaser K, Grundy E: **Marital status and long-term illness in Great Britain**. *Journal of Marriage and the Family* 1997:156-164.
12. Frisch M, Simonsen J: **Marriage, cohabitation and mortality in Denmark: national cohort study of 6.5 million persons followed for up to three decades (1982-2011)**. *Int J Epidemiol* 2013, **42**(2):559-578.
13. Scafato E, Galluzzo L, Gandin C, Ghirini S, Baldereschi M, Capurso A, Maggi S, Farchi G, For The Ilsa Working G: **Marital and cohabitation status as predictors of mortality: a 10-year follow-up of an Italian elderly cohort**. *Soc Sci Med* 2008, **67**(9):1456-1464.
14. Lund R, Due P, Modvig J, Holstein BE, Damsgaard MT, Andersen PK: **Cohabitation and marital status as predictors of mortality--an eight year follow-up study**. *Soc Sci Med* 2002, **55**(4):673-679.
15. Kilpi F, Konttinen H, Silventoinen K, Martikainen P: **Living arrangements as determinants of myocardial infarction incidence and survival: A prospective register study of over 300,000 Finnish men and women**. *Soc Sci Med* 2015, **133**:93-100.
16. Ross CE: **Reconceptualizing marital status as a continuum of social attachment**. *Journal of Marriage and the Family* 1995:129-140.
17. Koskinen S, Joutsenniemi K, Martelin T, Martikainen P: **Mortality differences according to living arrangements**. *Int J Epidemiol* 2007, **36**(6):1255-1264.
18. Staehelin K, Schindler C, Spoerri A, Zemp Stutz E, Swiss National Cohort Study G: **Marital status, living arrangement and mortality: does the association vary by gender?** *J Epidemiol Community Health* 2012, **66**(7):e22.
19. Franke S, Kulu H: **Mortality Differences by Partnership Status in England and Wales: The Effect of Living Arrangements or Health Selection?** *Eur J Popul* 2018, **34**(1):87-118.
20. Ikeda A, Iso H, Toyoshima H, Fujino Y, Mizoue T, Yoshimura T, Inaba Y, Tamakoshi A, Group JS: **Marital status and mortality among Japanese men and women: the Japan Collaborative Cohort Study**. *BMC Public Health* 2007, **7**(1):73.
21. Lillard LA, Waite LJ: **'Til death do us part: Marital disruption and mortality**. *American journal of sociology* 1995, **100**(5):1131-1156.
22. Gomez SL, Hurley S, Canchola AJ, Keegan TH, Cheng I, Murphy JD, Clarke CA, Glaser SL, Martinez ME: **Effects of marital status and economic resources on survival after cancer: A population-based study**. *Cancer* 2016, **122**(10):1618-1625.

For peer review only - http://bmjopen.bmj.com/site/about/guidelines.xhtml

23. Martikainen P, Valkonen T: **Mortality after the death of a spouse: rates and causes of death in a large Finnish cohort**. *Am J Public Health* 1996, **86**(8):1087-1093.
24. Honjo K, Iso H, Ikeda A, Inoue M, Sawada N, Tsugane S, Group JS: **Marital Transition and Risk of Stroke: How Living Arrangement and Employment Status Modify Associations**. *Stroke* 2016, **47**(4):991-998.
25. Jampaklay A, Haseen F: **MARITAL UNIONS AND UNMARRIED COHABITATION IN BANGKOK, THAILAND**. *Asian Population Studies* 2011, **7**(2):137-156.
26. Williams L, Kabamalan M, Ogena N: **Cohabitation in the Philippines: Attitudes and behaviors among young women and men**. *Journal of Marriage and Family* 2007, **69**(5):1244-1256.
27. **World Values Survey 2017 - last update: Online Data Analysis,** Available at: http://www.worldvaluessurvey.org/WVSOnline.jsp. [Accessed 10 Dec. 2017]
28. National Statistical Office (Thailand): **Population by religion, region and area, 2015**. 2016. http://web.nso.go.th/en/survey/popchan/data/2015-2016-Statistical%20tables%20PDF.pdf [Accessed 26 May 2021]
29. Saiwonsaroj K, Husa, K., Wohlschlagl, H. : **Fertility decline and the role of culture - Thailand's demographic challenges for the 21st Century**. In: *Southeast Asian Transformations.* Edited by Kurfürst SaW, S. Bielefeld: Transcript Verlag; 2020: 125-152.
30. Litalien M: **Social Inequalities and the Promotion of Women in Buddhism in Thailand**. *Journal of Buddhist Ethics* 2018, **25**:569-606.
31. Heuveline P, Hirschman C: **Fertility transition: Southeast Asia**. *International Encyclopedia of the Social and Behavioral Sciences* 2015, **9**:84-91.
32. Jones GW: **Changing marriage patterns in Asia**. In: *Routledge handbook of Asian demography.* Edited by Zhao Z, Hayes A: Routledge; 2017: 351-369.
33. Esara P: **Moral Scrutiny, Marriage Inequality: Cohabitation in Bangkok, Thailand**. *The Asia Pacific Journal of Anthropology* 2012, **13**(3):211-227.
34. National Statistical Office (Thailand): **Statistical Tables Population, Whole Kingdom**. 2007. http://web.nso.go.th/en/survey/pop_character/population%20characteristics%202005-2006/statistical%20tables%20%20population/ST1D36_wholekingdom.PDF [Accessed 21 Mar. 2018].
35. Federal Register of Legislation Australia: **Family Law Act 1975**. 2017-last update. Available at: https://www.legislation.gov.au/Details/C2017C00385 [Accessed 27 Dec. 2018].
36. Royal Government of Thailand: **Civil and Commercial Code**. In. Bangkok; 2015.
37. Lee KS, Ono H: **Marriage, cohabitation, and happiness: A cross - national analysis of 27 countries**. *Journal of Marriage and Family* 2012, **74**(5):953-972.
38. Soons JPM, Kalmijn M: **Is marriage more than cohabitation? Well - being differences in 30 European countries**. *Journal of Marriage and Family* 2009, **71**(5):1141-1157.
39. Holt-Lunstad J, Smith TB, Layton JB: **Social relationships and mortality risk: a meta-analytic review**. *PLoS Med* 2010, **7**(7):e1000316.
40. Seubsman SA, Yiengprugsawan V, Sleigh AC, the Thai Cohort Study T: **A large national Thai Cohort Study of the Health-Risk Transition based on Sukhothai Thammathirat Open University students**. *ASEAN J Open Distance Learn* 2012, **4**(1).
41. Sleigh AC, Seubsman SA, Bain C, Thai Cohort Study T: **Cohort profile: The Thai Cohort of 87,134 Open University students**. *Int J Epidemiol* 2008, **37**(2):266-272.
42. Prasartkul P, Vapattanawong P: **The completeness of death registration in Thailand: Evidence from demographic surveillance system of the Kanchanaburi Project**. *World Health Popul* 2006, **8**(3):43-51.
43. Hair JF, Anderson RE, Babin BJ, Black WC: **Multivariate data analysis: A global perspective: Pearson Upper Saddle River**. In.: NJ; 2010.
44. Shigetomi S: **Marriage and marriage registration in Thailand (<Special Issue>Focus on Asia)**. *Global Communication Studies* 2014(18 January 2019).
45. Diener E, Gohm CL, Suh E, Oishi S: **Similarity of the Relations between Marital Status and Subjective Well-Being Across Cultures**. *Journal of Cross-Cultural Psychology* 2000, **31**(4):419-436.
46. Gibbs JP: **Marital status and suicide in the United States: a special test of the status integration**

For peer review only - http://bmjopen.bmj.com/site/about/guidelines.xhtml

theory. *AJS* 1969, **74**(5):521-533.
47. Ji Y: **Asian Families at the Crossroads: A Meeting of East, West, Tradition, Modernity, and Gender**. *Journal of Marriage and Family* 2015, **77**(5):1031-1038.
48. Rindfuss RR, Bumpass LL, Choe MK, Tsuya NO: **Social networks and family change in Japan**. *American sociological review* 2004, **69**(6):838-861.
49. Kalmijn M: **The Influence of Men's Income and Employment on Marriage and Cohabitation: Testing Oppenheimer's Theory in Europe**. *Eur J Popul* 2011, **27**(3):269-293.
50. Marmot M: **The influence of income on health: views of an epidemiologist**. *Health Aff (Millwood)* 2002, **21**(2):31-46.
51. Hori M, Kamo Y: **Gender Differences in Happiness: the Effects of Marriage, Social Roles, and Social Support in East Asia**. *Applied Research in Quality of Life* 2018, **13**(4):839-857.
52. Robards J, Evandrou M, Falkingham J, Vlachantoni A: **Marital status, health and mortality**. *Maturitas* 2012, **73**(4):295-299.
53. National Statistical Office (Thailand): **The 2005 Health And Welfare Survey**. 2005. Available at: http://www.nso.go.th/sites/2014en/Pages/Statistical%20Themes/Population-Society/Social%20Security/Health--Welfare.aspx [Access 5 May 2021].
54. Mealing NM, Banks E, Jorm LR, Steel DG, Clements MS, Rogers KD: **Investigation of relative risk estimates from studies of the same population with contrasting response rates and designs**. *BMC Med Res Methodol* 2010, **10**:26.
55. Ponsonby AL, Dwyer T, Couper D: **Is this finding relevant? Generalisation and epidemiology**. *Aust N Z J Public Health* 1996, **20**(1):54-56.

For peer review only

Appendix

Appendix Table 1. Mortality Risk (Hazard Ratio) (2005–2016) for major covariates in the Thai Cohort Study, 2005-2016

Covariates	Category	Total	Male	Females
Sex	Males	Ref	--	--
	Females	0.52(0.45-0.61)***	--	--
Birth year	-1959	5.19(4.07-6.61)***	4.53(3.38-6.07)***	4.49(2.76-7.30)***
	1960-1969	2.27(1.84-2.80)***	1.77(1.36-2.31)***	3.16(2.25-4.45)***
	1970-1974	1.45(1.18-1.80)***	1.16(0.89-1.52)	1.99(1.40-2.81)***
	1975-1979	1.15(0.95-1.40)	0.98(0.77-1.25)	1.42(1.04-1.94)*
	1980-	Ref	Ref	Ref
Residence	Urban	Ref	Ref	Ref
	Rural	1.19(1.06-1.34)***	1.23(1.07-1.41)***	1.09(0.89-1.35)
Personal Income	-7000	Ref	Ref	Ref
	7001-20000	0.84(0.73-0.96)*	0.85(0.72-1.01)	0.82(0.65-1.04)
	20001-	0.62(0.50-0.76)***	0.59(0.46-.76)***	0.70(0.48-1.04)
Smoking status	Never	Ref	Ref	
	Former	1.19(1.01-1.40)*	1.22(1.02-1.46)*	0.92(0.56-1.49)
	current	1.84(1.55-2.18)***	1.82(1.52-2.18)***	2.69(1.47-4.93)***
Alcohol consumption	Occasional social drinker	Ref	Ref	Ref
	Never drinker	1.16(0.99-1.37)	1.14(0.88-1.47)	1.18(0.95-1.47)
	Current regular drinker	1.32(1.09-1.62)**	1.35(1.10-1.66)***	1.52(0.61-3.83)
	Now stopped	1.50(1.27-1.77)***	1.52(1.26-1.83)***	1.44(1.00-2.06)*
Pre-existing psychological conditions	Not Having	Ref	Ref	Ref
	Having	1.32(1.02-1.69)*	1.39(1.03-1.88)*	1.15(0.72-1.84)
Pre-existing physical conditions	Not having diabetes	Ref	Ref	Ref
	Having diabetes	2.40(1.83-3.15)***	2.32(1.71-3.13)***	3.42(1.89-6.20)***
	Not having cancer	Ref	Ref	Ref
	Having cancer	1.84(1.16-2.91)**	1.35(0.69-2.63)	2.70(1.44-5.06)***
	Not Having CVD	Ref	Ref	Ref
	Having CVD	1.36(0.87-2.11)	1.23(0.72-2.08)	2.30(1.07-4.96)*
	Not Having	Ref	Ref	Ref

For peer review only - http://bmjopen.bmj.com/site/about/guidelines.xhtml

	Kidney/liver diseases			
	Having Kidney/liver diseases	1.35(1.13-1.63)***	1.27(1.03-1.58)*	1.76(1.23-2.52)***
BMI	Underweight	1.08(0.88-1.31)	1.19(0.90-1.59)	1.05(0.80-1.39)
	Normal	Ref	Ref	Ref
	Overweight at risk	0.85(0.72-1.00)*	0.79(0.65-.95)*	1.10(0.80-1.53)
	Obese I	0.94(0.80-1.10)	0.88(0.74-1.06)	1.20(0.86-1.68)
	Obese II	1.27(0.96-1.68)	1.08(0.76-1.53)	1.85(1.15-2.96)*

For peer review only

For peer review only - http://bmjopen.bmj.com/site/about/guidelines.xhtml

Appendix Table 2. Adjusted mortality Risk (Hazard Ratio) (2005–2016) for exposure variables and major covariates, for all participants and excluding participants with pre-existing conditions in the Thai Cohort Study, 2005-2016

Covariates / Exposure variables	Category	Final model (N=79286)	Model excluding participants with pre-existed chronic health condition (N=70790)
Sex	Males	Ref	
	Females	0.52(0.45-.61)***	0.51(0.43-.60)***
Birth year	-1959	5.19(4.07-6.61)***	4.78(3.62-6.31)***
	1960-1969	2.27(1.84-2.80)***	2.27(1.80-2.86)***
	1970-1974	1.45(1.18-1.80)***	1.35(1.06-1.71)*
	1975-1979	1.15(0.95-1.40)	1.10(0.89-1.36)
	1980-	Ref	Ref
Residence	Urban	Ref	Ref
	Rural	1.19(1.06-1.34)***	1.14(1.00-1.29)
Personal Income	-7000	Ref	Ref
	7001-20000	0.84(0.73-.96)*	0.88(0.75-1.02)
	20001-	0.62(0.50-.76)***	0.60(0.47-.77)***
Smoking status	Never	Ref	
	Former	1.19(1.01-1.40)*	1.23(1.03-1.48)*
	current	1.84(1.55-2.18)***	1.85(1.53-2.25)***
Alcohol consumption	Occasional social drinker	Ref	
	Never drinker	1.16(0.99-1.37)	1.09(0.91-1.31)
	Current regular drinker	1.32(1.09-1.62)**	1.19(0.94-1.51)
	Now stopped	1.50(1.27-1.77)***	1.24(1.01-1.53)*
Pre-existing psychological conditions	Not Having	Ref	--
	Having	1.32(1.02-1.69)*	--
Pre-existing physical conditions	Not having diabetes	Ref	--
	Having diabetes	2.40(1.83-3.15)***	--
	Not having cancer	Ref	--
	Having cancer	1.84(1.16-2.91)**	--
	Not Having CVD	Ref	--

For peer review only - http://bmjopen.bmj.com/site/about/guidelines.xhtml

	Having CVD	1.36(0.87-2.11)	--
	Not Having Kidney/liver diseases	Ref	--
	Having Kidney/liver diseases	1.35(1.13-1.63)***	--
BMI	Underweight	1.08(0.88-1.31)	0.97(0.77-1.22)
	Normal	Ref	Ref
	Overweight at risk	0.85(0.72-1.00)*	0.86(0.72-1.04)
	Obese I	0.94(0.80-1.10)	0.98(0.82-1.18)
	Obese II	1.27(0.96-1.68)	1.67(1.23-2.26)***
**Having good family support**	Yes	Ref	Ref
	No	1.35(1.15-1.59)***	1.41(1.17-1.70)***
**Union Status**	Single	1.27(1.09-1.48)***	1.25(1.05-1.49)*
	Cohabited and living with a partner	1.39(1.06-1.83)*	1.49(1.10-2.02)**
	Cohabited but not living with a partner	0.96(0.52-1.76)	1.18(0.64-2.16)
	Married but not living with a partner	1.24(0.92-1.66)	1.37(0.99-1.90)
	Married and living with a partner	Ref	Ref
	Separated/divorced/widowed	1.46(1.13-1.87)***	1.63(1.23-2.16)***
	Separated/divorced/widowed but living with a partner	1.22(0.73-2.05)	0.94(0.47-1.90)

For peer review only - http://bmjopen.bmj.com/site/about/guidelines.xhtml

*Appendix Table 3. Mortality Risk (Hazard Ratio) (2005–2016) for union status and family support, by final unweighted model and weighted to age-sex structure of Thai national population *, persons aged 20-44 years, in the Thai Cohort Study, 2005-2016*

Variable	Category	Unweighted (N=71313)	Weighted by age-sex proportion of Thai population in 2005
Having good family support	Yes	Ref	Ref
	No	1.46(1.21-1.75)***	1.51(1.24-1.84)***
Union Status	Single	1.36(1.15-1.61)***	1.35(1.12-1.62)***
	Cohabited and living with a partner	1.52(1.14-2.03)***	1.47(1.09-1.98)*
	Cohabited but not living with a partner	1.04(0.57-1.91)	1.23(0.64-2.38)
	Married but not living with a partner	1.41(0.99-2.02)	1.59(1.08-2.33)*
	Married and living with a partner	Ref	Ref
	Separated/divorced/widowed	1.43(1.03-1.97)*	1.35(0.96-1.90)
	Separated/divorced/widowed but living with a partner	0.95(0.42-2.13)	0.88(0.37-2.08)

*from the Health and Welfare Survey 2005 conducted and reported by the Thai National Statistical Office (NSO)

For peer review only

For peer review only - http://bmjopen.bmj.com/site/about/guidelines.xhtml

Reporting checklist for cohort study.

Based on the STROBE cohort guidelines.

Instructions to authors

Complete this checklist by entering the page numbers from your manuscript where readers will find each of the items listed below.

Your article may not currently address all the items on the checklist. Please modify your text to include the missing information. If you are certain that an item does not apply, please write "n/a" and provide a short explanation.

Upload your completed checklist as an extra file when you submit to a journal.

In your methods section, say that you used the STROBE cohortreporting guidelines, and cite them as:

von Elm E, Altman DG, Egger M, Pocock SJ, Gotzsche PC, Vandenbroucke JP. The Strengthening the Reporting of Observational Studies in Epidemiology (STROBE) Statement: guidelines for reporting observational studies.

		Reporting Item	Page Number
**Title and abstract**			
Title	#1a	Indicate the study’s design with a commonly used term in the title or the abstract	3
Abstract	#1b	Provide in the abstract an informative and balanced summary of what was done and what was found	3-4
**Introduction**			
Background / rationale	#2	Explain the scientific background and rationale for the investigation being reported	6
Objectives	#3	State specific objectives, including any prespecified hypotheses	9
**Methods**			

For peer review only

Study design	#4	Present key elements of study design early in the paper	9-10
Setting	#5	Describe the setting, locations, and relevant dates, including periods of recruitment, exposure, follow-up, and data collection	10
Eligibility criteria	#6a	Give the eligibility criteria, and the sources and methods of selection of participants. Describe methods of follow-up.	10
Eligibility criteria	#6b	For matched studies, give matching criteria and number of exposed and unexposed	N/A
Variables	#7	Clearly define all outcomes, exposures, predictors, potential confounders, and effect modifiers. Give diagnostic criteria, if applicable	10-11
Data sources / measurement	#8	For each variable of interest give sources of data and details of methods of assessment (measurement). Describe comparability of assessment methods if there is more than one group. Give information separately for for exposed and unexposed groups if applicable.	10
Bias	#9	Describe any efforts to address potential sources of bias	5
Study size	#10	Explain how the study size was arrived at	10
Quantitative variables	#11	Explain how quantitative variables were handled in the analyses. If applicable, describe which groupings were chosen, and why	12
Statistical methods	#12a	Describe all statistical methods, including those used to control for confounding Page 12	
Statistical methods	#12b	Describe any methods used to examine subgroups and interactions	12
Statistical methods	#12c	Explain how missing data were addressed	N/a
Statistical methods	#12d	If applicable, explain how loss to follow-up was addressed	N/a
Statistical methods	#12e	Describe any sensitivity analyses	

For peer review only

**Results**			
Participants	#13a	Report numbers of individuals at each stage of study—eg numbers potentially eligible, examined for eligibility, confirmed eligible, included in the study, completing follow-up, and analysed. Give information separately for for exposed and unexposed groups if applicable.	10
Participants	#13b	Give reasons for non-participation at each stage	10
Participants	#13c	Consider use of a flow diagram	
Descriptive data	#14a	Give characteristics of study participants (eg demographic, clinical, social) and information on exposures and potential confounders. Give information separately for exposed and unexposed groups if applicable.	12
Descriptive data	#14b	Indicate number of participants with missing data for each variable of interest page 26	
Descriptive data	#14c	Summarise follow-up time (eg, average and total amount) page 10	
Outcome data	#15	Report numbers of outcome events or summary measures over time. Give information separately for exposed and unexposed groups if applicable. Page 13-15	
Main results	#16a	Give unadjusted estimates and, if applicable, confounder-adjusted estimates and their precision (eg, 95% confidence interval). Make clear which confounders were adjusted for and why they were included page 13-15	13-15
Main results	#16b	Report category boundaries when continuous variables were categorized	N/A

For peer review only

Main results	#16c	If relevant, consider translating estimates of relative risk into absolute risk for a meaningful time period	
Other analyses	#17	Report other analyses done—eg analyses of subgroups and interactions, and sensitivity analyses	13-15
**Discussion**			
Key results	#18	Summarise key results with reference to study objectives	15
Limitations	#19	Discuss limitations of the study, taking into account sources of potential bias or imprecision. Discuss both direction and magnitude of any potential bias.	5 and 18
Interpretation	#20	Give a cautious overall interpretation considering objectives, limitations, multiplicity of analyses, results from similar studies, and other relevant evidence.	18
Generalisability	#21	Discuss the generalisability (external validity) of the study results	18
**Other Information**			
Funding	#22	Give the source of funding and the role of the funders for the present study and, if applicable, for the original study on which the present article is based	2

None The STROBE checklist is distributed under the terms of the Creative Commons Attribution License CC-BY. This checklist can be completed online using https://www.goodreports.org/, a tool made by the EQUATOR Network in collaboration with Penelope.ai

For peer review only

How do cohabitation and marital status, affect mortality risk?: results from a cohort study in Thailand

Journal:	*BMJ Open*
Manuscript ID	bmjopen-2022-062811.R1
Article Type:	Original research
Date Submitted by the Author:	19-Jul-2022
Complete List of Authors:	Zhao, Jiaying; Australian National University<br>Law, CK; The University of Sydney<br>Kelly, Matthew; Australian National University, Department of Global Health<br>Yiengprugsawan, Vasoontara; University of New South Wales<br>Seubsman, Sam-ang; Sukhothai Thammathirat Open University<br>Sleigh, Adrian; Australian National University, National Centre for Epidemiology and Population Health
**Primary Subject Heading**:	Epidemiology
Secondary Subject Heading:	Public health
Keywords:	PUBLIC HEALTH, EPIDEMIOLOGY, SOCIAL MEDICINE

SCHOLARONE™ Manuscripts

I, the Submitting Author has the right to grant and does grant on behalf of all authors of the Work (as defined in the below author licence), an exclusive licence and/or a non-exclusive licence for contributions from authors who are: i) UK Crown employees; ii) where BMJ has agreed a CC-BY licence shall apply, and/or iii) in accordance with the terms applicable for US Federal Government officers or employees acting as part of their official duties; on a worldwide, perpetual, irrevocable, royalty-free basis to BMJ Publishing Group Ltd ("BMJ") its licensees and where the relevant Journal is co-owned by BMJ to the co-owners of the Journal, to publish the Work in this journal and any other BMJ products and to exploit all rights, as set out in our licence.

The Submitting Author accepts and understands that any supply made under these terms is made by BMJ to the Submitting Author unless you are acting as an employee on behalf of your employer or a postgraduate student of an affiliated institution which is paying any applicable article publishing charge ("APC") for Open Access articles. Where the Submitting Author wishes to make the Work available on an Open Access basis (and intends to pay the relevant APC), the terms of reuse of such Open Access shall be governed by a Creative Commons licence – details of these licences and which Creative Commons licence will apply to this Work are set out in our licence referred to above.

Other than as permitted in any relevant BMJ Author's Self Archiving Policies, I confirm this Work has not been accepted for publication elsewhere, is not being considered for publication elsewhere and does not duplicate material already published. I confirm all authors consent to publication of this Work and authorise the granting of this licence.

For peer review only

How do cohabitation and marital status, affect mortality risk?: results from a cohort study in Thailand

Jiaying Zhao[a], Chi Kin Law[b], Matthew Kelly[c§],Vasoontara Yiengprugsawan[d] , Sam-ang Seubsman[f], Adrian Sleigh[c]

[a]School of Demography, The Australian National University, Canberra, ACT, Australia 2601

[b]NHMRC Clinical Trials Centre, Faculty of Medicine and Health, The University of Sydney, Australia

[c]Department of Global Health, Research School of Population Health, The Australian National University, Australia

[d]Australian Research Council Centre of Excellence on Population Ageing Research, Business School, University of New South Wales, Australia

[f]School of Human Ecology, Sukhothai Thammathirat Open University, Pakkret, Nonthaburi, Thailand

[§] **Correspondence to:** Dr Matthew Kelly, 62 Mills Rd., The Australian National University, Acton 2601, Canberra, Australia. +61 2 6125 0714

Email Matthew.Kelly@anu.edu.au

Word count: 3921

For peer review only

Acknowledgements

We thank the staff at Sukhothai Thammathirat Open University (STOU) who assisted with student contact and the STOU students who are participating in the cohort study. We also thank Dr Bandit Thinkamrop and his team from Khon Kaen University for guiding us successfully through the complex data processing.

For peer review only

For peer review only - http://bmjopen.bmj.com/site/about/guidelines.xhtml

Cohabitation, marital status, mortality and gender difference in Thailand

Abstract

Objective

To examine the relationship between baseline union status (i.e. including marriage and cohabitation) and mortality, paying attention to gender differentials, through an 11-year follow-up of a large cohort in Thailand.

Design

Cohort data from Thai Cohort Study (TCS) were linked official death records over an 11 year follow-up period.

Setting

Community based adults in Thailand

Participants

87,151 Thai adults participated in TCS cohort.

Method Cox regression models measured longitudinal associations between union status and 11-year-mortality.

Results

For peer review only

For peer review only - http://bmjopen.bmj.com/site/about/guidelines.xhtml

From 2005 (baseline) to 2016, persons who cohabited and lived with a partner, married persons but not living with a partner, and separated /divorced/widowed people were more likely to die compared with those married and living together with a partner. Those who did not have good family support had a higher death risk than those having good family support.

Single or cohabiting women had higher risks of mortality than women who were married and living together with a partner throughout follow-up, while separated/divorced/widowed men had higher risks of mortality than counterpart males.

Conclusions

Our study reveals the protective effect of marriage and living together on mortality in Thailand, an understudied setting where institutionalization of cohabitation is low leading to a limited mortality protection. Public policies for moderating mortality should thus be gender nuanced, culturally and institutionally specific. Also, we demonstrate that in settings such as Thailand, where marital status is not always defined in the same way as in western cultures, the need to measure cohabitation in locally relevant terms is important.

Keywords: gender difference, cohabitation, marriage, mortality, Thailand

For peer review only - http://bmjopen.bmj.com/site/about/guidelines.xhtml

For peer review only

For peer review only - http://bmjopen.bmj.com/site/about/guidelines.xhtml

Strengths and Limitations of the study

- This is a large nationwide cohort (i.e. 87,151 participants) with 11-year follow-up period produced reliable estimate of effect sizes while also measuring the longitudinal effects of changes in union status on mortality.
- The large variety of variables collected in the study allow for control of multiple potential confounders.
- Cause of death was not able to be included in the analysis limiting the ability to interpret the findings
- The difficulty in defining marriage and cohabitation in Thailand may indicate there was some misclassification of individuals making the cohabitation – mortality relationship difficult to measure.

For peer review only

For peer review only - http://bmjopen.bmj.com/site/about/guidelines.xhtml

Introduction

Inequalities of mortality risk across categories of marital status have been well documented by previous longitudinal studies [1-3]. Married persons generally have lower mortality than those who are never married, divorced, separated, or widowed in both developed [4] and developing countries [5]. Such patterns were found to be more marked among men than women [6, 7].

Two major processes have been proposed to explain the marital status differences in mortality risks: (1) selection mechanisms and; (2) protection mechanisms [1, 8]. Selection theory suggests that unhealthy people ("confounding variable") may be selected outside the bounds of marriage into other union types, such as cohabitation [9]. The 'Protection mechanisms' theory suggests that marital status has a causal impact on mortality risk reduction through the provision of social/ economic support, through improvement of unhealthy behaviours and mental health ("intermediate variable"), and provision of health information [1, 3, 6, 10, 11]. A meta-analysis indicates that support from family was more beneficial than support provided by friends in reducing mortality [6]. A further clarification of the interaction between family support and union status and their effects on mortality can improve our understanding of the pathways between union status and mortality risk reduction.

Cohabitation may facilitate similar functions as marriage, though such a protection effect may be weaker than marriage and may vary across populations [9, 12, 13]. Some evidence from developed countries shows that cohabiting persons have lower mortality than those living alone, particularly for men and a combination of married and cohabited state may better predict mortality than marriage alone [13, 14]. Similar to married status, cohabitation may improve health through emotional and instrumental support from partners [15]. But cohabitation, with lower levels of commitment, support and stability than marriage, could constrain economic, social, and psychological resources [9, 15, 16]. Also, living arrangements, incorporating the partnership situation and household composition, was

For peer review only - http://bmjopen.bmj.com/site/about/guidelines.xhtml
For peer review only

associated with mortality [17, 18] . Evidence on the health effect of cohabitation, particularly incorporating living arrangements is inconsistent and sparse in developing countries.

Gender differences could be another potential moderator for the effect of union status on mortality [3, 14]. Men's mortality may profit more from union status than women [3, 13, 19, 20]. Men may benefit more from risk behaviour reduction and emotional support than women [19, 21], while women may benefit more financially [1, 22]. After union disillusion, excess mortality is greater among men than women [20, 23]. This may be because women are more likely to adopt the role of providing emotional support and performing household tasks for their spouse. Men may suffer more once union status dissolves [13] but separated/divorced/ widowed women may feel relieved of their emotional duty to care, and consequently lower their mortality [24].

Gender differences in the effect of union status on mortality may vary in societies with different gender role norms (e.g. the male breadwinner model) [24]. In societies with a low degree of institutionalization of cohabitation, the mortality benefit from union status due to financial support may be weaker for cohabited women than married women. Cohabited women may also benefit less emotionally from union status due to low social acceptance of cohabitation.

Cohabitation has become more common in Asian countries [25, 26]. In Thailand, the proportion of cohabiting individuals increased from 6% in 2005-2009 to 9% in 2010-2014 [27]. However, cohabitation may have different social, cultural and institutional interpretations as well as legal rights for relationships in Eastern societies.

Thai society has strong religious identification [28], and more traditional cultural norms around cohabitation [29]. The strong religious identification has a role in the production of gender norms and expectations [30]. However, during the past decades, attitudes towards marriage have been changing [31, 32] . Increased female workforce participation, work related pressures, high costs of living and

For peer review only - http://bmjopen.bmj.com/site/about/guidelines.xhtml

an increasing burden of care giving for elderly parents have led to delayed marriages and downward pressure on marriage rates [31, 33].

Reported cohabitation rates remained low in Thailand, however this may relate to the inexact definition of marriage in Thai society. State registration of marriage is practiced in Thailand but those married through traditional Buddhist religious ceremonies are also considered married. In 2005, around 34% of currently married people were not registered, and the proportion was higher for those aged 15-34 years [34]. Overall, cohabiting without marriage in Thailand is likely to be more common than surveys reveal [31]. Younger, more educated and Bangkok dwelling Thais are more likely to cohabit with their partner [25].

In many Western countries de facto partnering confers rights and obligations on partners that are identical to those married. Those rights include economic security and sharing of marital property in the event of divorce [35]. However, cohabitation in Thailand does not incur the same legal outcomes as registered marriages in terms of sharing property and other assets [36].

Variation in different social, cultural and institutional interpretations as well as legal rights for relationships may lead to different associations between cohabitation and mortality [6, 37, 38]. The gap in mental health between married and cohabitating individuals is larger in countries with a low degree of institutionalization of cohabitation. In such countries the following features are expected: 1) cohabitation is relatively uncommon and low in social acceptance [38]; 2) traditional gender role persists [37]; 3) and religiosity is high [37]. Disapproval of cohabitations and uncertainty of future may lead to lower level of social support and poorer mental wellbeing for cohabiters in such society [38].

Previous studies examined the influence of marital status on mortality in Eastern societies but did not differentiate between cohabitation and marriage [39]. It remains unclear whether cohabitation affords Eastern populations' protective benefits similar to marriage.

For peer review only - http://bmjopen.bmj.com/site/about/guidelines.xhtml

For peer review only

This study aims to fill this knowledge gap by examining the relationship between baseline union status (i.e. including marriage and cohabitation) and mortality, paying attention to gender differentials, through an 11-year follow-up of a large cohort in Thailand. The potential mechanism linking union status to mortality from socioeconomic, psychological, and behavioural aspects will be investigated.

Hypotheses

Considering the institutional and cultural features around marriage in Thailand, we test the following hypotheses:

1. People who were single, or separated/divorced/widowed have higher mortality than married people living together.

2. Cohabiting people living with a partner have a higher mortality risk than married people living together, but may have lower mortality than single, or separated/divorced/widowed people.

3. People who have stronger family support have lower mortality, and this effect cannot be fully explained by differentials in union status.

4. 'Separated/divorced/widowed' males have a higher mortality risk compared with married men living together with a partner, while such higher mortality risk may be moderated among females.

5. Cohabiting women living with a partner have a higher mortality risk compared with married women living together with a partner, while such higher mortality risk may be limited among males

Methods

Study population and study design

For peer review only - http://bmjopen.bmj.com/site/about/guidelines.xhtml

Data from a large nationwide longitudinal study of health dynamics in Thailand – the Thai Cohort Study (TCS) – were linked with official death records over an 11 year follow-up period. The baseline cohort was recruited in 2005 with a questionnaire mailed to all current students of Sukhothai Thammathirat Open University (STOU); 87,151 responded, covering 43.5% of the STOU students at that time. STOU students are off-campus adult distance-learners residing throughout the country. Characteristics of TCS members are similar to the overall Thai population, in terms of social geography, religion, socioeconomic status, and income, but members are in general more highly educated and more urban [40]. In 2005, at the study baseline, the cohort and the general Thai population shared a median age of 29 years, median income of around $2500USD, were 94% Buddhist, and just over 50% female, and had a similar regional distribution across the country [40, 41].

Measurements

Mortality

Mortality status was traced with death registration records from the Thai Ministry of Interior covering the period from TCS baseline in 2005 to November 2016. The current death registration system covers about 95% of all deaths in Thailand [42]. Data linkage was conducted by matching the unique citizen identification number, which cohort members provided to the study at baseline. By November 2016, 1401 deaths were recorded within the cohort.

Union status

Using the TCS baseline data, union status was categorised by the following three questions: 1. ‘What is your current status?’ (single, living with partner, or married). Those who responded ‘Married’ were further classified by question：2.’What is your current marital status?’ Those who responded ‘Married – first and only marriage’ or ‘Remarried – second or later marriage’

For peer review only

were classified as ‘Married’, while those who responded ‘Separated from someone you have been married to (but not divorced)’, ‘Divorced’, or ‘Widowed’ were classified as ‘Separated/divorced/widowed’. We further classified union status with living arrangement by 3. ‘Do any of the following people (i.e. Spouse / partner) usually live in your home?’

Conjugal union status was coded into seven categories: (1) Single, (2) cohabited and living with a partner, (3) cohabited but not living with a partner, (4) married but not living together with a partner, (5) married and living together with a partner (the reference group), (6) separated/divorced/widowed, and (7) separated/divorced/widowed but living with a partner.

Family support

Family support was assessed by the following question using a four-point scale: ‘How would you rate the support you are getting from your family?’ The proportion of deaths during 11 year follow up for those reporting ‘quite a bit of support’, and ‘a lot of support’ was 1.65% and 1.45%, while that for those reporting ‘‘very little’, ‘little’, or ‘Not relevant’ was 2.67%, 2.24% and 3.75% respectively. The statistical power may be influenced due to the small number of deceased participants for those with little (128), very little (57) support and not relevant (23), and a merger of the three groups enables a more robust statistical analysis in the present study. Therefore, those who responded ‘very little’, ‘little’, or ‘Not relevant’ were coded as not having good family support, while those who responded ‘quite a bit of support’, and ‘a lot of support’ were coded as having good family support.

Other baseline covariates

A wide range of covariates were available including baseline variables describing sex, birth year, urban or rural residence, and income, smoking status and alcohol consumption at baseline,

For peer review only - http://bmjopen.bmj.com/site/about/guidelines.xhtml

baseline pre-existing psychological and physical health conditions and baseline BMI. Details of each covariate are shown in Table 1.

We also tested social support in the model. Social support was measured using the question ‘How would you rate the support you are getting from the following: government, religious groups, and good friends/neighbours/ people in workplace’. Those who responded ‘very little’ or ‘little’, ‘not relevant’ were classified as not having good social support for that question. But these covariates were not significantly associated with mortality; and we did not report it in the final result.

For peer review only

Table 1 The measurements and classification of *baseline (2005) covariates*

**Variable**	**Measurement Question**	**Original response category**	**Analytical category**
Sex	You are:-	Male; Female	Male; Female
Birth year	In what year were you born?	Year of birth	-1959; 1960-1969; 1970-1974; 1975-1979; 1980-
Residence	Where is your current permanent home located now?	Countryside; City/Town	Urban; Rural
Income	What is your average personal monthly income?	less than 3,000 Baht; 3,001-7,000 Baht;<br>7,001-10,000 Baht; 10,001-20,000 Baht ; 20,001-30,000 Baht;<br>over 30,000 Baht	Personal monthly income: ≤7,000, 7001-20,000, or >20,000 Baht (1 $US ~ 30 Baht)
Smoking status	1.Have you ever smoked?;<br>2. If you have **quit** smoking, at what age did you stop?	1. Yes; No (will categorised 'Never').<br>2. XX years; still smoking (will be categorised as 'current smoker'), otherwise will be categorised as 'former smoker',	Never; Former smoker; Current smoker
Alcohol consumption	Have you ever drunk alcohol?	Occasional social drinker; No, never;<br>Current regular drinker;<br>Used to drink before, now	Never drinker; Occasional social drinker; current regular drinker; former drinker

For peer review only - http://bmjopen.bmj.com/site/about/guidelines.xhtml

		stopped	
Pre-existing physical conditions	The next questions ask about some health conditions whether you ever been told by a doctor that you have this condition. (please tick all that apply)	Diabetes (needing insulin); Diabetes (do not need insulin); Liver disease (not cancer); Kidney disease; High cholesterol/high blood lipids; High blood pressure; Ischemic (coronary) heart disease; Cerebrovascular disease (stroke); liver cancer; lung cancer; Cancer of the digestive system; Breast cancer; Other cancers	Whether a doctor had ever diagnosed: diabetes, cancer, cardiovascular diseases (CVD), kidney and liver diseases
Pre-existing psychological conditions	The next questions ask about some health conditions whether you ever been told by a doctor that you have this condition. (please tick all that apply)	Depression / anxiety	Whether a doctor had ever diagnosed psychological conditions (i.e. depression or anxiety)
Body mass index (BMI)	1.What is your weight?<br>2. What is your height without shoes?	1. XXX kilograms<br>2. XXX cm	Calculated by validated self-reported height and weight (Lim et al. 2009). Asian BMI standards: underweight (<18.5), normal (18.5 to <23), overweight at risk (23 to <25), obese I (25 to <30), and obese II (≥30) (Kanazawa et al. 2002)

For peer review only - http://bmjopen.bmj.com/site/about/guidelines.xhtml

Statistical analyses

Our longitudinal analyses of all-cause mortality continued until the end of the observation period in November 2016. The exposure variables of interest were union status and family support. We tabulated November 2016 survival status by union status, family support and all covariates.

We examined links between mortality as the outcome and union status and family support as the mutually adjusted exposure variables of interest. We used Cox regression to estimate multivariate hazard ratios (HR) for men and women and their 95% confidence intervals (CI) given the assumption of proportionality of hazards were held. Covariates were included in sequence as models were developed incrementally.

To examine the effect of other covariates on the association between baseline union status and the subsequent mortality outcome, 4 regression models were compiled. The included covariates and the exposure variables of interest for each model are shown in Table 2. The variance inflation factors (VIF) for all covariates and the exposure variables were less than 4, indicating no multicollinearity issues in the models [43]. We further applied the above four models by sex.

From Model 1 to Model 3, we gradually added confounding variables at individual, family and socio-economic levels. In Model 4, we included health behaviour and pre-existing psychological and physical conditions and BMI. These health behaviour and status variables may serve as "intermediating factors" for the effect of union status on mortality if the HR for union status changed significantly when these variables were added in the Model. If HR for union status changed little, these health behaviour and status variables may act as confounding variables for the effect of union status on mortality [44].

Table 2 Variables included in the analysed models

Model	Variables included
1	baseline union status and demographic variables (i.e. sex, year of birth, residence)
2	variables in Model 1, and family support

For peer review only - http://bmjopen.bmj.com/site/about/guidelines.xhtml

3	variables in Model 2, and socio-economic variables (i.e. income)
4	variables in Model 3, and related health behaviours (i.e. smoking status, alcohol consumption), pre-existing psychological and physical conditions, and BMI

Patient and public involvement statement

Study participants were all enrolled students at an Open University in Thailand. Research questions and approaches were first piloted with these students and the study design was adjusted accordingly. At various stages through the research project participants were provided with summary results of the study. As well the lead investigator made frequent public appearances on University radio and TV media to discuss the results of the study.

Results

At 2005 baseline, 49.3% of cohort members were single, 4.9% were cohabited and living with a partner, 1.4% were cohabited but not living with a partner, 3% were married but not living with a partner, 35.3% were married living together with a partner, and 3.7% were separated /divorced/widowed and not living with a partner, 0.6% were separated /divorced/widowed but living with a partner. There were 89.7% members reporting have good family support. The relationship between baseline union status, family support, and a range of potential confounders were shown in Table 3.

For peer review only - http://bmjopen.bmj.com/site/about/guidelines.xhtml

Table 3 *Socio-demographic attributes at baseline (2005) by union status in the Thai Cohort Study*

		Single (%)		Cohabited and living with a partner (%)		Cohabited but not living with a partner (%)		Married but not living with a partner (%)		Married and living with a partner (%)		Separated/ divorced/ widowed (%)		Separated/ divorced/ widowed but living with a partner (%)		Missing (%)		Total (%)	
Total		42931		4235		1201		2624		30721		3206		566		1667		87151	
Sex	Males	16649	38.8	1983	46.8	517	43.0	1340	51.1	16759	54.6	1086	33.9	345	61.0	806	48.4	39485	45.3
	Females	26282	61.2	2252	53.2	684	57.0	1284	48.9	13962	45.4	2120	66.1	221	39.0	853	51.2	47658	54.7
	Missing	0	0.0	0	0.0	0	0.0	0	0.0	0	0.0	0	0.0	0	0.0	8	0.5	8	0.0
Birth year	-1959	584	1.4	48	1.1	10	0.8	304	11.6	3799	12.4	460	14.3	107	18.9	74	4.4	5386	6.2
	1960-1969	3404	7.9	399	9.4	76	6.3	732	27.9	11596	37.7	1353	42.2	260	45.9	217	13.0	18037	20.7
	1970-1974	5248	12.2	715	16.9	149	12.4	610	23.2	7454	24.3	735	22.9	120	21.2	243	14.6	15274	17.5
	1975-1979	12604	29.4	1434	33.9	464	38.6	680	25.9	5831	19.0	513	16.0	60	10.6	423	25.4	22009	25.3
	1980-	21088	49.1	1639	38.7	502	41.8	298	11.4	2035	6.6	144	4.5	19	3.4	702	42.1	26427	30.3
	Missing	3	0.0	0	0.0	0	0.0	0	0.0	6	0.0	1	0.0	0	0.0	8	0.5	18	0.0
Residence	Urban	21571	50.2	2486	58.7	700	58.3	1241	47.3	15867	51.6	1732	54.0	359	63.4	808	48.5	44764	51.4
	Rural	21098	49.1	1729	40.8	495	41.2	1359	51.8	14621	47.6	1427	44.5	199	35.2	820	49.2	41748	47.9
	Missing	262	0.6	20	0.5	6	0.5	24	0.9	233	0.8	47	1.5	8	1.4	39	2.3	639	0.7
Personal Income	-7000	23166	54.0	2053	48.5	541	45.0	818	31.2	6966	22.7	1019	31.8	140	24.7	933	56.0	35636	40.9
	7001-20000	16543	38.5	1810	42.7	587	48.9	1446	55.1	17513	57.0	1633	50.9	294	51.9	542	32.5	40368	46.3
	20001-	1927	4.5	275	6.5	50	4.2	321	12.2	5752	18.7	450	14.0	110	19.4	71	4.3	8956	10.3
	Missing	1295	3.0	97	2.3	23	1.9	39	1.5	490	1.6	104	3.2	22	3.9	121	7.3	2191	2.5
Smoking status	Never	33659	78.4	2863	67.6	809	67.4	1648	62.8	19035	62.0	2135	66.6	291	51.4	712	42.7	61152	70.2
	Former	4950	11.5	732	17.3	224	18.7	570	21.7	7181	23.4	583	18.2	169	29.9	293	17.6	14702	16.9
	current	3550	8.3	567	13.4	148	12.3	293	11.2	3533	11.5	359	11.2	90	15.9	192	11.5	8732	10.0
	Missing	772	1.8	73	1.7	20	1.7	113	4.3	972	3.2	129	4.0	16	2.8	470	28.2	2565	2.9
Alcohol consumption	Occasional social drinker	24676	57.5	2648	62.5	831	69.2	1607	61.2	18463	60.1	1877	58.5	345	61.0	924	55.4	51371	58.9
	Never drinker	13181	30.7	902	21.3	182	15.2	559	21.3	6818	22.2	715	22.3	84	14.8	271	16.3	22712	26.1
	Current regular drinker	1243	2.9	251	5.9	65	5.4	144	5.5	2187	7.1	173	5.4	54	9.5	54	3.2	4171	4.8
	Now stopped	3416	8.0	404	9.5	114	9.5	267	10.2	2848	9.3	387	12.1	78	13.8	210	12.6	7724	8.9
	Missing	415	1.0	30	0.7	9	0.7	47	1.8	405	1.3	54	1.7	5	0.9	208	12.5	1173	1.3
Pre-existing psychological conditions	Not Having	41365	96.4	4083	96.4	1157	96.3	2546	97.0	29875	97.2	3013	94.0	536	94.7	1585	95.1	84160	96.6
	Having	1566	3.6	152	3.6	44	3.7	78	3.0	846	2.8	193	6.0	30	5.3	82	4.9	2991	3.4

For peer review only - http://bmjopen.bmj.com/site/about/guidelines.xhtml

Union status, family support, and mortality

From 2005 (baseline) to 2016, separated /divorced/widowed people and married not living with a partner were more likely to die than married people living with a partner , but singles and those who cohabited were less likely to die compared to married people living together with a partner (Table 4). Those not having good family support were more likely to die than those having family support. Males, older birth cohorts, those living in rural areas, current smokers, current regular drinkers, those having psychological and chronic physical disease, and obese people were more likely to die.

For peer review only

For peer review only - http://bmjopen.bmj.com/site/about/guidelines.xhtml

Table 4. Survival status (%) by sex, union status, family support, and potential confounders for the Thai Cohort Study, 2005-2016

		Males		Females		Total	
		Survival Status on December 201					
		Alive (%)	Dead (%)	Alive (%)	Dead (%)	Alive (%)	Dead (%)
	Number of death	38507	978	47235	423	85750	1401
Birth year	-1959	93.0	7.0	97.9	2.1	94.6	5.4
	1960-1969	97.3	2.7	98.6	1.4	97.9	2.1
	1970-1974	98.1	1.9	99.1	0.9	98.6	1.4
	1975-1979	98.3	1.7	99.3	0.7	98.9	1.1
	1980-	98.2	1.8	99.4	0.6	99.0	1.0
	Missing	100.0	0.0	100.0	0.0	100.0	0.0
Residence	Urban	97.7	2.3	99.1	0.9	98.5	1.5
	Rural	97.3	2.7	99.1	0.9	98.3	1.7
	Missing	95.7	4.3	99.4	0.6	97.7	2.3
Personal Income	-7000	97.6	2.4	99.2	0.8	98.6	1.4
	7001-20000	97.7	2.3	99.1	0.9	98.4	1.6
	20001-	97.2	2.8	98.8	1.2	97.9	2.1
	Missing	95.1	4.9	98.9	1.1	97.2	2.8
Smoking status	Never	98.2	1.8	99.1	0.9	98.9	1.1
	Former	97.3	2.7	99.0	1.0	97.6	2.4
	current	96.4	3.6	97.5	2.5	96.5	3.5
	Missing	96.4	3.6	99.1	0.9	98.1	1.9
Alcohol consumption	Occasional social drinker	97.9	2.1	99.2	0.8	98.5	1.5
	Never drinker	97.8	2.2	99.1	0.9	98.8	1.2
	Current regular drinker	96.6	3.4	98.1	1.9	96.7	3.3
	Now stopped	95.8	4.2	98.8	1.2	97.1	2.9
	Missing	96.2	3.8	99.4	0.6	98.2	1.8
Pre-existing psychological conditions	Not Having Psychological Diseases	97.6	2.4	99.1	0.9	98.4	1.6
	Having Psychological Diseases	96.0	4.0	98.7	1.3	97.6	2.4
Pre-existing physical conditions	Not having diabetes	97.6	2.4	99.1	0.9	98.5	1.5

For peer review only - http://bmjopen.bmj.com/site/about/guidelines.xhtml
For peer review only

	Having diabetes	90.5	9.5	94.6	5.4	91.7	8.3
	Not having cancer	97.5	2.5	99.1	0.9	98.4	1.6
	Having cancer	94.0	6.0	95.9	4.1	95.2	4.8
	Not Having CVD	97.6	2.4	99.1	0.9	98.4	1.6
	Having CVD	92.7	7.3	96.6	3.4	94.4	5.6
	Not Having Kidney/liver Diseases	97.6	2.4	99.2	0.8	98.5	1.5
	Having Kidney/liver Diseases	96.3	3.7	98.0	2.0	97.0	3.0
BMI	Underweight(<18.5)	97.3	2.7	99.2	0.8	98.9	1.1
	Normal (18.5 to <23)	97.7	2.3	99.2	0.8	98.6	1.4
	Overweight at risk (23 to <25)	97.8	2.2	98.9	1.1	98.2	1.8
	Obese I (25 to <30)	97.3	2.7	98.8	1.2	97.8	2.2
	Obese II (⩾30)	96.5	3.5	97.9	2.1	97.2	2.8
	Missing	94.9	5.1	98.5	1.5	96.5	3.5
**Having good family support**	Yes	97.6	2.4	99.2	0.8	98.5	1.5
	No	96.5	3.5	98.6	1.4	97.5	2.5
	Missing	96.9	3.1	99.6	0.4	98.2	1.8
**Union status**	Single	98.0	2.0	99.2	0.8	98.7	1.3
	Cohabited and living with a partner	97.9	2.1	98.9	1.1	98.5	1.5
	Cohabited but not living with a partner	98.5	1.5	99.6	0.4	99.1	0.9
	Married but not living with a partner	96.7	3.3	98.7	1.3	97.7	2.3
	Married and living with a partner	97.3	2.7	99.1	0.9	98.1	1.9
	Separated/divorced/widowed	94.3	5.7	98.8	1.2	97.3	2.7
	Separated/divorced/widowed but living with a partner	95.7	4.3	98.2	1.8	96.6	3.4
	Missing	97.3	2.7	98.7	1.3	98.0	2.0

For peer review only

In the Cox proportional hazard model (Table 5), after controlling for sex, birth cohort, rural or urban residence, singles, persons who cohabited and living with a partner, married persons but not living with a partner, and separated /divorced/widowed people were more likely to die compared with those married living together with a partner (Model 1). Further adjustment of family support (Model 2) reduced the corresponding HR. Those who do not have good family support had a higher death risk than those having good family support. Overall, no significant interaction was found between union status and family support ($p>0.05$) (Appendix Table 1 and 2). We stratified by whether participants had good family support at baseline. Among respondents who had poor family support, no mortality risk difference was found in those cohabited and living with a partner compared with those who married living together with a partner.

For peer review only - http://bmjopen.bmj.com/site/about/guidelines.xhtml

Table 5. *Gender difference, Union status (2005)* [§]*, family support (2005), and Mortality (2005–2016) in the Thai Cohort Study, 2005-2016*

	Sex	Number of cases included in the model (% of total sample by sex)	Union Status						Not Having Good Family Support
			Single	cohabited and living with partner	Cohabit but not living with partner	Married but not living together	Separated, divorce or widowed	Separated, Divorced, widowed, but living with partner	
1		84874 (97.4%)	1.40(1.21-1.61)***	1.50(1.15-1.96)***	0.98(0.53-1.78)	1.37(1.05-1.79)*	1.73(1.38-2.18)***	1.46(0.92-2.34)	--
2		84444 (96.9%)	1.36(1.17-1.57)***	1.46(1.12-1.90)**	0.95(0.52-1.74)	1.35(1.03-1.76)*	1.65(1.31-2.08)***	1.39(0.87-2.22)	1.39(1.19-1.62)***
3		82461 (94.6%)	1.28(1.11-1.49)***	1.43(1.09-1.87)*	0.95(0.52-1.73)	1.27(0.97-1.67)	1.48(1.16-1.88)***	1.16(0.70-1.95)	1.38(1.18-1.61)***
4	Total	79286 (91.0%)	1.27(1.09-1.48)***	1.39(1.06-1.83)*	0.96(0.52-1.76)	1.24(0.92-1.66)	1.46(1.13-1.87)***	1.22(0.73-2.05)	1.35(1.15-1.59)***
1		38390 (97.2%)	1.36(1.13-1.63)***	1.34(0.96-1.87)	1.05(0.52-2.12)	1.32(0.97-1.80)	2.18(1.67-2.85)***	1.39(0.82-2.37)	--
2		38169 (96.7%)	1.32(1.09-1.58)***	1.30(0.93-1.82)	1.03(0.51-2.09)	1.29(0.94-1.77)	2.09(1.59-2.74)***	1.33(0.78-2.27)	1.30(1.08-1.57)**
3		37325 (94.5%)	1.20(0.99-1.45)	1.23(0.88-1.73)	0.99(0.49-2.01)	1.19(0.86-1.64)	1.87(1.41-2.48)***	1.04(0.57-1.90)	1.30(1.08-1.57)**
4	Males	35907 (90.9%)	1.17(0.95-1.42)	1.17(0.83-1.65)	0.99(0.49-2.02)	1.14(0.80-1.62)	1.79(1.33-2.40)***	1.11(0.61-2.03)	1.29(1.06-1.57)**
1		46484 (97.5%)	1.42(1.11-1.81)**	1.87(1.19-2.94)**	0.81(0.26-2.58)	1.51(0.90-2.54)	1.12(0.72-1.73)	1.78(0.66-4.82)	--
2		46275 (97.1%)	1.39(1.09-1.78)**	1.80(1.15-2.82)*	0.79(0.25-2.49)	1.49(0.88-2.50)	1.07(0.69-1.66)	1.65(0.61-4.47)	1.60(1.21-2.10)***
3		45136 (94.7%)	1.40(1.10-1.80)**	1.87(1.19-2.93)**	0.83(0.26-2.63)	1.52(0.90-2.56)	0.98(0.62-1.55)	1.70(0.63-4.61)	1.56(1.18-2.06)***
4	Females	43379 (91.0%)	1.40(1.08-1.81)**	1.87(1.19-2.96)**	0.88(0.28-2.78)	1.49(0.87-2.56)	0.99(0.62-1.59)	1.75(0.64-4.75)	1.52(1.14-2.03)***

Note:
Model 1 included union status+age+sex+urban (family support not included)
Model 2 included union status+family support+age+sex+urban
Model 3 included union status+family support+age+sex+urban+income
Model 4 included union status+family support+age+sex+urban+income+ health behaviour +doctor diagnosed depression or anxiety+pre-existing physical conditions

For peer review only - http://bmjopen.bmj.com/site/about/guidelines.xhtml

§Reference group: married people and living together
* p<0.05
**p<0.01
***p<0.005

For peer review only

When the effect of personal income was included (Model 3), the HR reduced but remained significant for singles, cohabited and living with a partner, and separated /divorced/widowed people, but the HR for those married but not living with a partner became not significant. HR for family support did not change significantly when income was included. When health behaviours, pre-existing psychological conditions and chronic conditions (Model 4) were included, the HR for union status and family support changed little.

In the final Model 4 certain covariates also associated with mortality risk were included. Males, older birth cohort members, rural residents, persons with low income, smokers, current regular drinkers or those who stopped drinking, and those having psychological or physical conditions were all more likely to die, compared with their counterparts (Appendix Table 1)

Sex differentials in the effect of union status on mortality

We repeated the models by sex with results shown in Table 5. Males and females had a different mortality differential pattern by union status. After controlling for birth cohort, rural or urban residence, both single men and women had higher risks of dying than their counterparts who have married living together with a partner. However, cohabited women living with a partner had higher risks of dying than married women living together with a partner, while such increase in mortality risk was not significant among men. By contrast, separated /divorced/widowed men had a higher mortality risk than counterpart men, while such risk did not significantly increase among separated /divorced/widowed women (Model 1). Further adjustment for family support (Model 2) reduced the corresponding HR. The HR for those not having good family support for females was higher than males.

When income was included in Model 3, the HRs for single and separated /divorced/widowed men reduced, but remained similar for single women. The HRs for cohabited women living with a partner was similar to those in Model 2, while those for men became not significant. HR for family

For peer review only - http://bmjopen.bmj.com/site/about/guidelines.xhtml

support did not change significantly when income was included. In the final Model 4, when health behaviours, pre-existing psychological and chronic conditions and BMI were included HR for union status and family support changed little in these models.

In the final Model 4, single women, cohabited women living with a partner, and separated/divorce/widowed men had significantly higher mortality risks than their married counterparts living together with a partner.

Effect of union status on mortality by age group

A further subgroup analysis by age group was conducted (see Appendix Table 2). Overall, results of this subgroup analysis were in line with the main results. The cohabited group had a higher risk of dying among the younger age group (HR=1.54, p<0.05), when compared with the reference group. On the contrary, the higher risk of dying for persons who were separated, divorce or widowed was appeared in the older age group (HR=1.45, p<0.005). Results of this analysis were generally in line with the results among whole sample.

Discussion

The analyses add to Eastern population evidence on links between union status and mortality risk, as well as considering the under-explored factor of cohabitation. Single or cohabiting women had higher risks of mortality than women who were married and living together with a partner throughout follow-up. However, such increasing risk for single or cohabiting men was moderate. Separated/divorce/widowed men also had higher risks of mortality than men who were married and living together with a partner. Family support acts as a protective factor against mortality but such protective effect cannot fully be attributed to union status. These results confirm most of the hypotheses we proposed, except that cohabiting people do not appear to experience any protective effect over those who were single. In regard to the protective effect of marriage on mortality, this analysis agrees with much of the literature, and with similar effect sizes. For example in a meta-

For peer review only - http://bmjopen.bmj.com/site/about/guidelines.xhtml

analysis, relative risks for mortality were calculated for widowed (1.11), divorced/separated (1.16) and never married (1.11) individuals, respectively [1]. Other studies however have found that cohabiting has a similar protective effect to marriage, unlike this Thai study. In fact in several studies there was no independent effect found for marriage on mortality, the protective function appeared to result from living together in any union status [13, 14]. More complex analyses which take into account a range of factors around social interaction appear to predict mortality more accurately than models that use union status alone [39].

A possible explanation for this divergence in results, may lie with the particular cultural setting of Thailand, and the lack of studies in similar settings. Defining and measuring marital status and cohabitation rates has historically been quite difficult in Thailand [32]. Buddhist society has not traditionally had a definite view on when couples are married and this has led to legal issues surrounding inheritance, property and alimony. Generally a man's obligation to treat a woman as his wife was defined through the payment of a dowry by the woman's family, without legal registration [45].

A Family Registration Act in 1935 attempted to formalise marriage registration [45]. However, in modern Thailand multiple combinations of religious ceremony and state registration are still generally recognised [33]; and the Thai census itself may classify as married individuals who have only performed ceremonial bonding [31]. This is pertinent to our study with the possibility that some of those who self-reported being married may in fact according to western definitions be living in a cohabiting relationship. In Thailand cohabiting remains legally distinguishable from marriage and does not appear to confer mortality benefit but this needs more focused research to explain our results.

A potential explanation of high mortality risk for single and cohabited women living with a partner may be a consequence of social stigmatisation towards single or cohabitation status [46, 47]. Social stigma towards single and cohabited persons and consequent stress of not being married may

For peer review only - http://bmjopen.bmj.com/site/about/guidelines.xhtml
For peer review only

adversely affect persons' wellbeing and health status, in a society with a high prevalence of marriage [46]. In a society where most individuals are married, unmarried individuals have been seen as deviant from social role expectations [46]. For women, a 'marriage package' even comes laden with more traditional expectations of and for women [48, 49]. Behaving against the norm may cause single and cohabitated people to evaluate their lives as more incomplete than married persons [46]. As well as this potential negative effect of not marrying, the converse is also possible. Marrying can have positive effects in terms of the instrumental and emotional support provided through marriage, and the improved sense of belonging [50].

Results of our analysis showed that the mortality risk differentials for single males reduced and were no longer significant once income was included in the model. In contrast, the same adjustment had a limited impact for women. There is some evidence that men's low economic position would deter union formation, and that effect was more influential in countries where gender roles are more traditional [51]. Low-income men who are unable to fulfil the role of breadwinner are not only less attractive for marriage [51], but their low socioeconomic status also leads to a higher risk of mortality [52]. Further, in a traditional society (like Thailand), low-income men who failed to meet the social norm of 'male breadwinner' may suffer from higher psychological distress [53].

The gender difference in effect of marriage dissolution is also noteworthy. Separated/divorced/widowed men had much higher mortality risk than women. This result has also been found in previous studies, with the differential reducing in older age groups; that is middle-aged divorced or widowed men have the highest relative mortality risk [6, 54]. One proposed mechanism for this differential is a greater reduction in social ties and social support experienced by men when marriages end [3]. With women being more responsible for maintaining friendship and family ties they are more capable to maintain these relationships after the marriage ends [3].

The analysis included living arrangement combined with union status. It showed those married but living separately with a partner generally have a higher risk than those were married and living

For peer review only - http://bmjopen.bmj.com/site/about/guidelines.xhtml

together, though the effect is not statistically significant in the final model. It is possible that those who died during 2005-2016 were ill or at greater risks of mortality at baseline. There were 9353 participants (10.7% of cohort) reporting having physician's diagnosis of depression / anxiety, cancer, cardiovascular disease, diabetes, liver disease, or kidney diseases at baseline. We performed a sensitivity analysis by excluding these participants from the survival analysis (see Appendix Table 2) and found HRs for union status and family support were generally similar to the final model (Model 4), which has adjusted for participants' baseline disease profile.

There were 79,295 (91% among 87,151) cohort members aged from 20-44 years. A sensitivity analysis was conducted to compare the final model (Model 4) among those aged 20-44 years with the model weighted by nationally representative Thai population. The national data were derived from the Health and Welfare Survey 2005 conducted and reported by the National Statistical Office (Thailand) [54]. The HRs for union status and family support between the original model and model weighted by the Thai population were generally similar (Appendix Table 3).

Our large nationwide cohort and extensive follow-up period produced reliable estimate of effect sizes while also measuring the longitudinal effects of changes in union status. Although the TCS participants are not randomly representative in terms of educational attainment, the majority Buddhist religion, geographical distribution, variance in socio-economic wellbeing, as well as their community embedded residences give a reasonable picture of union status and mortality in Thailand [40]. The TCS is also future focused, including aspirational self-motivated Thais with increasing levels of education. Furthermore, unlike cross-sectional studies, longitudinal cohort analyses are designed to investigate causal relationships between exposures and outcomes within the same group of cohort population over time. Selection bias when establishing the cohort should not interfere with relative risk estimates between exposed and unexposed individuals for the socio-biological outcome of death [55]. HRs estimated from groups within a cohort remain valid and can be generalised more broadly [56, 57]. In addition, the difficulty in defining marriage and

For peer review only - http://bmjopen.bmj.com/site/about/guidelines.xhtml

cohabitation in Thailand may indicate there was some misclassification of individuals making the cohabitation – mortality relationship difficult to measure.

The study also has some limitations. First, for those who not having good family support, the mechanism for the effect of cohabitation on mortality may be different. Among those not having good family support, no significant mortality risk difference was found in those cohabited and living with a partner compared with the reference group (i.e. who married living together with a partner). With limited support from family, cohabited individuals may face comparably less pressure from social norms but enjoy benefit (e.g. emotional and financial support) from the relationship [45]. However, due to the small number of deaths (n=) from this specified group, we did not explore further analysis. Also we also cannot be certain that the 'family support' variable does not also measure support from spouses or partners as well as other family members, though the questionnaire does use the Thai word implying wider family.

Second, we only have cause of death (COD) data for 583 deceased participants from 2005 to December 2010, accounting for 41.6% of all deaths. However, given the limited sample size (e.g. only 78 CVD deaths, 118 neoplasms deaths, and 204 injury deaths), it is not sufficient to assess the statistical association between union status and mortality by cause of death in the present analysis. Further analysis could be conducted in future research.

Third, we are unable to test the temporal alignments between the marriage status and the onset of the health condition that led to mortality. This means we cannot identify more precisely at what point in disease progression marital status exerts an influence.

Although this paper reports on data collected in Thailand, the results do have broader significance. Across Southeast Asia similar trends in changes in marriage and cohabitation are being observed. Female labour force participation and higher education participation along with changes in societal values are driving delayed marriage and increased cohabitation without marriage region wide [58,

For peer review only - http://bmjopen.bmj.com/site/about/guidelines.xhtml

59]. This means that the associations we have measured here will have implications for other countries in the region experiencing similar transitions.

Conclusions

Our 11-year cohort study revealed the protective effect of marriage on mortality in transitional Southeast Asia, an understudied Eastern setting. But such a protective effect of cohabitation, particularly for women, was limited in the Thai context. The gender difference found in this analysis suggests that union status effect on mortality may be mediated by socio-cultural factors in a society, which deserves further investigation. The need to measure marriage and cohabitation in institutionally and culturally relevant terms is important. Formulation and implementation of public policies for moderating mortality should be gender nuanced and culturally and institutionally specific. The correct policy will respond to the current situation and encourage people to be aware of the potential effect of union status on health and wellbeing.

Thailand, as with most Southeast and East Asian countries, is undergoing a transition towards increased singlehood, delayed marriage, decreased family sizes and an ageing population. The findings of this paper in terms of the protective effect of marriage on health then have important implications for consideration of policies to promote healthy ageing. Healthy ageing policies cannot promote marriage, but can be sensitive to the increasing number of single and cohabiting individuals and their increased mortality risk.

Abbreviations

BMI: Body mass index; CI: Confidence Interval; CVD: cardiovascular diseases; HR: hazard ratios; TCS: Thai Cohort Study.

For peer review only

Author Contributions

JZ and CKL conceived the study design and led data analysis, MK and VY led data interpretation and manuscript writing. AS and SS conceived the overall study and made substantial contributions to data interpretation and critically reviewing the manuscript. All authors contributed to review and approval of the final draft.

Funding statement

This study was supported by the International Collaborative Research Grants Scheme with joint grants from the Wellcome Trust UK (GR071587MA) and the Australian NHMRC (268055) and by a global health grant from the NHMRC (585426).

Competing interests

All authors declare no conflict of interest in this paper

Ethical approval

Informed written consent was obtained from all participants. All students were advised that they could withdraw, or not participate, without any effect on their academic progress. The questionnaires never sought sensitive personal information and no biological samples were taken. Ethics approval was obtained from Sukhothai Thammathirat Open University Research and Development Institute (protocol 0522/10) and the Australian National University Human Research Ethics Committee (protocols 2004/344 and 2009/570).

Data availability statement

Data from the Thai Cohort Study are available upon reasonable request from the corresponding author (MK).

For peer review only

References:

1. Manzoli L, Villari P, G MP, Boccia A: **Marital status and mortality in the elderly: a systematic review and meta-analysis**. *Soc Sci Med* 2007, **64**(1):77-94.
2. Lund R, Holstein BE, Osler M: **Marital history from age 15 to 40 years and subsequent 10-year mortality: a longitudinal study of Danish males born in 1953**. *Int J Epidemiol* 2004, **33**(2):389-397.
3. Shor E, Roelfs DJ, Bugyi P, Schwartz JE: **Meta-analysis of marital dissolution and mortality: reevaluating the intersection of gender and age**. *Soc Sci Med* 2012, **75**(1):46-59.
4. Ebrahim S, Wannamethee G, McCallum A, Walker M, Shaper AG: **Marital status, change in marital status, and mortality in middle-aged British men**. *Am J Epidemiol* 1995, **142**(8):834-842.
5. Hurt LS, Ronsmans C, Saha S: **Effects of education and other socioeconomic factors on middle age mortality in rural Bangladesh**. *J Epidemiol Community Health* 2004, **58**(4):315-320.
6. Shor E, Roelfs DJ, Yogev T: **The strength of family ties: A meta-analysis and meta-regression of self-reported social support and mortality**. *Social Networks* 2013, **35**(4):626-638.
7. Tatangelo G, McCabe M, Campbell S, Szoeke C: **Gender, marital status and longevity**. *Maturitas* 2017, **100**:64-69.
8. Joung IM, van de Mheen HD, Stronks K, van Poppel FW, Mackenbach JP: **A longitudinal study of health selection in marital transitions**. *Soc Sci Med* 1998, **46**(3):425-435.
9. Liu H, Reczek C: **Cohabitation and US adult mortality: An examination by gender and race**. *Journal of Marriage and Family* 2012, **74**(4):794-811.
10. Cheung YB: **Marital status and mortality in British women: a longitudinal study**. *Int J Epidemiol* 2000, **29**(1):93-99.
11. Murphy M, Glaser K, Grundy E: **Marital status and long-term illness in Great Britain**. *Journal of Marriage and the Family* 1997:156-164.
12. Frisch M, Simonsen J: **Marriage, cohabitation and mortality in Denmark: national cohort study of 6.5 million persons followed for up to three decades (1982-2011)**. *Int J Epidemiol* 2013, **42**(2):559-578.
13. Scafato E, Galluzzo L, Gandin C, Ghirini S, Baldereschi M, Capurso A, Maggi S, Farchi G, For The Ilsa Working G: **Marital and cohabitation status as predictors of mortality: a 10-year follow-up of an Italian elderly cohort**. *Soc Sci Med* 2008, **67**(9):1456-1464.
14. Lund R, Due P, Modvig J, Holstein BE, Damsgaard MT, Andersen PK: **Cohabitation and marital status as predictors of mortality--an eight year follow-up study**. *Soc Sci Med* 2002, **55**(4):673-679.
15. Kilpi F, Konttinen H, Silventoinen K, Martikainen P: **Living arrangements as determinants of myocardial infarction incidence and survival: A prospective register study of over 300,000 Finnish men and women**. *Soc Sci Med* 2015, **133**:93-100.
16. Ross CE: **Reconceptualizing marital status as a continuum of social attachment**. *Journal of Marriage and the Family* 1995:129-140.
17. Koskinen S, Joutsenniemi K, Martelin T, Martikainen P: **Mortality differences according to living arrangements**. *Int J Epidemiol* 2007, **36**(6):1255-1264.
18. Staehelin K, Schindler C, Spoerri A, Zemp Stutz E, Swiss National Cohort Study G: **Marital status, living arrangement and mortality: does the association vary by gender?** *J Epidemiol Community Health* 2012, **66**(7):e22.
19. Franke S, Kulu H: **Mortality Differences by Partnership Status in England and Wales: The Effect of Living Arrangements or Health Selection?** *Eur J Popul* 2018, **34**(1):87-118.
20. Ikeda A, Iso H, Toyoshima H, Fujino Y, Mizoue T, Yoshimura T, Inaba Y, Tamakoshi A, Group JS: **Marital status and mortality among Japanese men and women: the Japan Collaborative Cohort Study**. *BMC Public Health* 2007, **7**(1):73.
21. Lillard LA, Waite LJ: **'Til death do us part: Marital disruption and mortality**. *American journal of sociology* 1995, **100**(5):1131-1156.
22. Gomez SL, Hurley S, Canchola AJ, Keegan TH, Cheng I, Murphy JD, Clarke CA, Glaser SL, Martinez ME: **Effects of marital status and economic resources on survival after cancer: A population-based study**. *Cancer* 2016, **122**(10):1618-1625.

For peer review only

23. Martikainen P, Valkonen T: **Mortality after the death of a spouse: rates and causes of death in a large Finnish cohort**. *Am J Public Health* 1996, **86**(8):1087-1093.
24. Honjo K, Iso H, Ikeda A, Inoue M, Sawada N, Tsugane S, Group JS: **Marital Transition and Risk of Stroke: How Living Arrangement and Employment Status Modify Associations**. *Stroke* 2016, **47**(4):991-998.
25. Jampaklay A, Haseen F: **MARITAL UNIONS AND UNMARRIED COHABITATION IN BANGKOK, THAILAND**. *Asian Population Studies* 2011, **7**(2):137-156.
26. Williams L, Kabamalan M, Ogena N: **Cohabitation in the Philippines: Attitudes and behaviors among young women and men**. *Journal of Marriage and Family* 2007, **69**(5):1244-1256.
27. **World Values Survey 2017 - last update: Online Data Analysis,** Available at: http://www.worldvaluessurvey.org/WVSOnline.jsp. [Accessed 10 Dec. 2017]
28. National Statistical Office (Thailand): **Population by religion, region and area, 2015**. 2016. http://web.nso.go.th/en/survey/popchan/data/2015-2016-Statistical%20tables%20PDF.pdf [Accessed 26 May 2021]
29. Saiwonsaroj K, Husa, K., Wohlschlagl, H. : **Fertility decline and the role of culture - Thailand's demographic challenges for the 21st Century**. In: *Southeast Asian Transformations.* Edited by Kurfürst SaW, S. Bielefeld: Transcript Verlag; 2020: 125-152.
30. Litalien M: **Social Inequalities and the Promotion of Women in Buddhism in Thailand**. *Journal of Buddhist Ethics* 2018, **25**:569-606.
31. Heuveline P, Hirschman C: **Fertility transition: Southeast Asia**. *International Encyclopedia of the Social and Behavioral Sciences* 2015, **9**:84-91.
32. Jones GW: **Changing marriage patterns in Asia**. In: *Routledge handbook of Asian demography.* Edited by Zhao Z, Hayes A: Routledge; 2017: 351-369.
33. Esara P: **Moral Scrutiny, Marriage Inequality: Cohabitation in Bangkok, Thailand**. *The Asia Pacific Journal of Anthropology* 2012, **13**(3):211-227.
34. National Statistical Office (Thailand): **Statistical Tables Population, Whole Kingdom**. 2007. http://web.nso.go.th/en/survey/pop_character/population%20characteristics%202005-2006/statistical%20tables%20%20population/ST1D36_wholekingdom.PDF [Accessed 21 Mar. 2018].
35. Federal Register of Legislation Australia: **Family Law Act 1975**. 2017-last update. Available at: https://www.legislation.gov.au/Details/C2017C00385 [Accessed 27 Dec. 2018].
36. Royal Government of Thailand: **Civil and Commercial Code**. In. Bangkok; 2015.
37. Lee KS, Ono H: **Marriage, cohabitation, and happiness: A cross‐national analysis of 27 countries**. *Journal of Marriage and Family* 2012, **74**(5):953-972.
38. Soons JPM, Kalmijn M: **Is marriage more than cohabitation? Well‐being differences in 30 European countries**. *Journal of Marriage and Family* 2009, **71**(5):1141-1157.
39. Holt-Lunstad J, Smith TB, Layton JB: **Social relationships and mortality risk: a meta-analytic review**. *PLoS Med* 2010, **7**(7):e1000316.
40. Seubsman SA, Yiengprugsawan V, Sleigh AC, the Thai Cohort Study T: **A large national Thai Cohort Study of the Health-Risk Transition based on Sukhothai Thammathirat Open University students**. *ASEAN J Open Distance Learn* 2012, **4**(1).
41. Sleigh AC, Seubsman SA, Bain C, Thai Cohort Study T: **Cohort profile: The Thai Cohort of 87,134 Open University students**. *Int J Epidemiol* 2008, **37**(2):266-272.
42. Prasartkul P, Vapattanawong P: **The completeness of death registration in Thailand: Evidence from demographic surveillance system of the Kanchanaburi Project**. *World Health Popul* 2006, **8**(3):43-51.
43. Hair JF, Anderson RE, Babin BJ, Black WC: **Multivariate data analysis: A global perspective: Pearson Upper Saddle River**. In.: NJ; 2010.
44. Baron R, Kenny, D: **The moderator-mediator variable distinction in social psychological research: Conceptual, strategic, and statistical considerations**. *Journal of Personality and Social Psychology*, 51, 1173-1182
45. Shigetomi S: **Marriage and marriage registration in Thailand (<Special Issue>Focus on Asia)**. *Global Communication Studies* 2014(18 January 2019).

For peer review only - http://bmjopen.bmj.com/site/about/guidelines.xhtml

46. Diener E, Gohm CL, Suh E, Oishi S: **Similarity of the Relations between Marital Status and Subjective Well-Being Across Cultures**. *Journal of Cross-Cultural Psychology* 2000, **31**(4):419-436.
47. Gibbs JP: **Marital status and suicide in the United States: a special test of the status integration theory**. *AJS* 1969, **74**(5):521-533.
48. Ji Y: **Asian Families at the Crossroads: A Meeting of East, West, Tradition, Modernity, and Gender**. *Journal of Marriage and Family* 2015, **77**(5):1031-1038.
49. Rindfuss RR, Bumpass LL, Choe MK, Tsuya NO: **Social networks and family change in Japan**. *American sociological review* 2004, **69**(6):838-861.
50. Zhu H, Danan G: **The protective effect of marriage on health and survival: does it persist at oldest-old ages?** *Journal of Population and Ageing*, 2010, 3:161-182.
51. Kalmijn M: **The Influence of Men's Income and Employment on Marriage and Cohabitation: Testing Oppenheimer's Theory in Europe**. *Eur J Popul* 2011, **27**(3):269-293.
52. Marmot M: **The influence of income on health: views of an epidemiologist**. *Health Aff (Millwood)* 2002, **21**(2):31-46.
53. Hori M, Kamo Y: **Gender Differences in Happiness: the Effects of Marriage, Social Roles, and Social Support in East Asia**. *Applied Research in Quality of Life* 2018, **13**(4):839-857.
54. Robards J, Evandrou M, Falkingham J, Vlachantoni A: **Marital status, health and mortality**. *Maturitas* 2012, **73**(4):295-299.
55. National Statistical Office (Thailand): **The 2005 Health And Welfare Survey**. 2005. Available at: http://www.nso.go.th/sites/2014en/Pages/Statistical%20Themes/Population-Society/Social%20Security/Health--Welfare.aspx [Access 5 May 2021].
56. Mealing NM, Banks E, Jorm LR, Steel DG, Clements MS, Rogers KD: **Investigation of relative risk estimates from studies of the same population with contrasting response rates and designs**. *BMC Med Res Methodol* 2010, **10**:26.
57. Ponsonby AL, Dwyer T, Couper D: **Is this finding relevant? Generalisation and epidemiology**. *Aust N Z J Public Health* 1996, **20**(1):54-56.
58. Yeung WJ, Desai S, Jones GW: **Families in Southeast and South Asia**. *Annual Review in Sociology.* 2018, 44:469-495.
59. Raymo JM, Park H, Xie Y, Yeung WJ: **Marriage and family in East Asia: continuity and change**. *Annual Review in Sociology*. 2015, 41:8.1-8.22.

For peer review only

Appendix

Appendix Table 1 Union status (2005) §, family support (2005), and Mortality (2005–2016) by family support in the Thai Cohort Study, 2005-2016

Model§		Union Status							
		Single	cohabited and living with partner	Cohabit but not living with partner	Married but not living together	Married and living together	Separated, divorce or widowed	Separated, Divorced, widowed, but living with partner	Not Having Good Family Support
Model 2		1.36(1.17-1.57)***	1.46(1.12-1.90)**	0.95(0.52-1.74)	1.35(1.03-1.76)*	Reference	1.65(1.31-2.08)***	1.39(0.87-2.22)	1.39(1.19-1.62)***
Model 2.1		1.31(1.12-1.53)***	1.64(1.24-2.17)***	0.81(0.40-1.64)	1.32(0.99-1.77)	Reference	1.49 (1.13-1.98)**	1.57 (0.92-2.67)	0.85(0.28-2.58)
	Interaction term	1.83 (0.59-5.70）	0.49（0.11-2.21）	3.33 (0.59-18.81)	1.72(0.46-6.24)	1.49 (0.47-4.67）	2.15(0.65-7.11)	--	--
Model 2.2		1.31(1.12-1.53)***	1.64(1.24-2.17)***	0.81(0.40-1.63)	1.32(0.98-1.76)	Reference	1.50 (1.14-1.99)***	1.58 (0.93-2.68)	--
Model 2.3		1.61(1.12-2.34)*	0.55(0.20-1.55)	1.91(0.59-6.19)	1.60(0.79-3.24)	Reference	2.09(1.34-3.26)***	1.09 (0.40-3.01)	--

§Model 2: included union status+family support+age+sex+urban

Model 2.1: included union status+family support+age+sex+urban+union status*family support

Model 2.2 for those with good family support only: included union statust+age+sex+urban

Model 2.3 for those without good family support only: included union statust+age+sex+urban

* p<0.05

**p<0.01

***p<0.005

For peer review only

Appendix Table 2 Union status (2005) §, family support (2005), and Mortality (2005–2016) by age group in the Thai Cohort Study, 2005-2016

Birth year		Number of cases included in the model (% of total sample by sex)	Union Status						Not Having Good Family Support
			Single	cohabited and living with partner	Cohabit but not living with partner	Married but not living together	Separated, divorce or widowed	Separated, Divorced, widowed, but living with partner	
-1974		84874 (97.4%)	1.40(1.21-1.61)***	1.50(1.15-1.96)***	0.98(0.53-1.78)	1.37(1.05-1.79)*	1.73(1.38-2.18)***	1.46(0.92-2.34)	--
1975-		35181(90.9%)	1.17(0.98-1.43)	1.18(0.77-1.81)	1.60(0.71-3.59)	1.33(0.97-1.82)	1.45(1.12-1.89)***	1.10(0.63-1.91)	1.38(1.14-1.68)***
Total		44104(91.1%)	1.31(0.98-1.74)	1.54(1.02-2.31)*	0.64(0.26-1.60)	0.78(0.34-1.81)	1.01(0.40-2.52)	2.58(0.62-10.60)	1.31(0.99-1.75)

Models included union status+family support+age+sex+urban+income+ health behaviour +doctor diagnosed depression or anxiety+pre-existing physical conditions

§Reference group: married people and living together

* p<0.05
**p<0.01
***p<0.005

For peer review only

Appendix Table 3 Number of death by cause of death and union status in the Thai Cohort Study, 2005- 2010

Cause of Death*	Union Status								
	Single	cohabited and living with partner	Cohabit but not living with partner	Married but not living together	Married living together	Separated, divorce or widowed	Separated, Divorced, widowed, but living with partner	Missing	Total
Cardiovascular disease	29 (37.18%)	5 (6.41%)	0 (0%)	7 (8.97%)	33 (42.31%)	2 (2.56%)	0 (0%)	2 (2.56%)	78 (100%)
Neoplasms	33 (27.97%)	1（0.85%)	0 (0%)	8 (6.78%)	57 (48.31%)	15 (12.71%)	1 (0.85%)	3 (2.54%)	118 (100%)
Injury	107 (52.45%)	12 (5.88%)	3 (1.47%)	4 (1.96%)	67 (32.84%)	10 (4.90%)	0 (0%)	1 (0.49%)	204(100%)
Other	78 (42.62%)	5 (2.73%)	2 (1.09%)	11 (6.01%)	69 (37.70%)	8 (4.37%)	3 (1.64%)	7 (3/83%)	183 (100%)

*Cardiovascular disease (CVD) (ICD code = I00-I99), Neoplasms (ICD code = C00-D48), injury (ICD code = V01-Y98), and from “other causes of death”

For peer review only

Reporting checklist for cohort study.

Based on the STROBE cohort guidelines.

Instructions to authors

Complete this checklist by entering the page numbers from your manuscript where readers will find each of the items listed below.

Your article may not currently address all the items on the checklist. Please modify your text to include the missing information. If you are certain that an item does not apply, please write "n/a" and provide a short explanation.

Upload your completed checklist as an extra file when you submit to a journal.

In your methods section, say that you used the STROBE cohortreporting guidelines, and cite them as:

von Elm E, Altman DG, Egger M, Pocock SJ, Gotzsche PC, Vandenbroucke JP. The Strengthening the Reporting of Observational Studies in Epidemiology (STROBE) Statement: guidelines for reporting observational studies.

		Reporting Item	Page Number
**Title and abstract**			
Title	#1a	Indicate the study's design with a commonly used term in the title or the abstract	3
Abstract	#1b	Provide in the abstract an informative and balanced summary of what was done and what was found	3-4
**Introduction**			
Background / rationale	#2	Explain the scientific background and rationale for the investigation being reported	6
Objectives	#3	State specific objectives, including any prespecified hypotheses	9
**Methods**			

For peer review only

Study design	#4	Present key elements of study design early in the paper	9-10
Setting	#5	Describe the setting, locations, and relevant dates, including periods of recruitment, exposure, follow-up, and data collection	10
Eligibility criteria	#6a	Give the eligibility criteria, and the sources and methods of selection of participants. Describe methods of follow-up.	10
Eligibility criteria	#6b	For matched studies, give matching criteria and number of exposed and unexposed	N/A
Variables	#7	Clearly define all outcomes, exposures, predictors, potential confounders, and effect modifiers. Give diagnostic criteria, if applicable	10-11
Data sources / measurement	#8	For each variable of interest give sources of data and details of methods of assessment (measurement). Describe comparability of assessment methods if there is more than one group. Give information separately for for exposed and unexposed groups if applicable.	10
Bias	#9	Describe any efforts to address potential sources of bias	5
Study size	#10	Explain how the study size was arrived at	10
Quantitative variables	#11	Explain how quantitative variables were handled in the analyses. If applicable, describe which groupings were chosen, and why	12
Statistical methods	#12a	Describe all statistical methods, including those used to control for confounding Page 12	
Statistical methods	#12b	Describe any methods used to examine subgroups and interactions	12
Statistical methods	#12c	Explain how missing data were addressed	N/a
Statistical methods	#12d	If applicable, explain how loss to follow-up was addressed	N/a
Statistical methods	#12e	Describe any sensitivity analyses	

For peer review only

**Results**			
Participants	#13a	Report numbers of individuals at each stage of study—eg numbers potentially eligible, examined for eligibility, confirmed eligible, included in the study, completing follow-up, and analysed. Give information separately for for exposed and unexposed groups if applicable.	10
Participants	#13b	Give reasons for non-participation at each stage	10
Participants	#13c	Consider use of a flow diagram	
Descriptive data	#14a	Give characteristics of study participants (eg demographic, clinical, social) and information on exposures and potential confounders. Give information separately for exposed and unexposed groups if applicable.	12
Descriptive data	#14b	Indicate number of participants with missing data for each variable of interest page 26	
Descriptive data	#14c	Summarise follow-up time (eg, average and total amount) page 10	
Outcome data	#15	Report numbers of outcome events or summary measures over time. Give information separately for exposed and unexposed groups if applicable. Page 13-15	
Main results	#16a	Give unadjusted estimates and, if applicable, confounder-adjusted estimates and their precision (eg, 95% confidence interval). Make clear which confounders were adjusted for and why they were included page 13-15	13-15
Main results	#16b	Report category boundaries when continuous variables were categorized	N/A

For peer review only

Main results	#16c	If relevant, consider translating estimates of relative risk into absolute risk for a meaningful time period	
Other analyses	#17	Report other analyses done—eg analyses of subgroups and interactions, and sensitivity analyses	13-15
**Discussion**			
Key results	#18	Summarise key results with reference to study objectives	15
Limitations	#19	Discuss limitations of the study, taking into account sources of potential bias or imprecision. Discuss both direction and magnitude of any potential bias.	5 and 18
Interpretation	#20	Give a cautious overall interpretation considering objectives, limitations, multiplicity of analyses, results from similar studies, and other relevant evidence.	18
Generalisability	#21	Discuss the generalisability (external validity) of the study results	18
**Other Information**			
Funding	#22	Give the source of funding and the role of the funders for the present study and, if applicable, for the original study on which the present article is based	2

None The STROBE checklist is distributed under the terms of the Creative Commons Attribution License CC-BY. This checklist can be completed online using https://www.goodreports.org/, a tool made by the EQUATOR Network in collaboration with Penelope.ai

For peer review only
